# Sickle Eye Project: a cross-sectional, non-interventional study of the prevalence of visual impairment due to sickle cell retinopathy and maculopathy in the UK

**DOI:** 10.1136/bmjopen-2023-082471

**Published:** 2024-02-28

**Authors:** Christiana Dinah, Konstantinos Balaskas, Brigit Greystoke, Rossby Awadzi, Peter Beke, Roger Ahern, James Talks

**Affiliations:** 1 Ophthalmology, London North West Healthcare NHS Trust, Harrow, UK; 2 Moorfields Eye Hospital NHS Foundation Trust, London, UK; 3 Institute of Ophthalmology, UCL, London, UK; 4 Royal Victoria Infirmary, Newcastle upon Tyne, UK; 5 London North West University Healthcare NHS Trust, Harrow, UK; 6 Lived experience expert, London, UK; 7 Independent Statistician, London, UK; 8 Newcastle Upon Tyne Hospitals NHS Foundation Trust, Newcastle Upon Tyne, UK

**Keywords:** Medical retina, Anaemia, Health Services

## Abstract

**Introduction:**

Sickle cell disease (SCD) is one of the most common genetic disorders in the UK, with over 15 000 people affected. Proliferative sickle cell retinopathy (SCR) is a well-described complication of SCD and can result in significant sight loss, although the prevalence in the UK is not currently known. There are currently no national screening guidelines for SCR, with wide variations in the management of the condition across the UK.

**Methods and analysis:**

The Sickle Eye Project is an epidemiological, cross-sectional, non-interventional study to determine the prevalence of visual impairment due to SCR and/or maculopathy in the UK. Haematologists in at least 16 geographically dispersed hospitals in the UK linked to participating eye clinics will offer study participation to consecutive patients meeting the inclusion criteria attending the sickle cell clinic. The following study procedures will be performed: (a) best corrected visual acuity with habitual correction and pinhole, (b) dilated slit lamp biomicroscopy and funduscopy, (c) optical coherence tomography (OCT), (d) OCT angiography where available, (e) ultrawide fundus photography, (f) National Eye Institute Visual Function Questionnaire-25 and (g) acceptability of retinal screening questionnaire. The primary outcome is the proportion of people with SCD with visual impairment defined as logarithm of the minimum angle of resolution ≥0.3 in at least one eye. Secondary outcomes include the prevalence of each stage of SCR and presence of maculopathy by age and genotype; correlation of stage of SCR and maculopathy to severity of SCD; the impact of SCR and presence of maculopathy on vision-related quality of life; and the acceptability to patients of routine retinal imaging for SCR and maculopathy.

**Ethics and dissemination:**

Ethical approval was obtained from the South Central–Oxford A Research Ethics Committee (REC 23/SC/0363). Findings will be reported through academic journals in ophthalmology and haematology.

**STRENGTHS AND LIMITATIONS OF THIS STUDY**

- The Sickle Eye Project will involve a large, geographically diverse random sample of people with sickle cell disease (SCD) attending routine haematology clinics, which is likely to be a representative sample of the SCD population in the UK.
- The study will assess the correlation between surrogates of severity of SCD and retinal imaging biomarkers.
- The study will use modern retinal imaging and clinical examination, in addition to incorporating double grading by expert graders and retinal specialists at Moorfields Reading Centre.
- One limitation is that the prevalence data and the imaging findings pertain to people with SCD in the UK and may not be directly generalisable to other health systems, especially Africa and Asia, which are the continents with the highest prevalence of SCD.

## Background

### Proliferative sickle cell retinopathy

Sickle cell disease (SCD) is one of the most common genetic disorders in the UK, with approximately 15 000 people affected.^1^ It is a debilitating, multisystem disease, associated with episodes of acute illness, progressive organ damage and reduced life expectancy. There are 13 genetic variants of SCD, with HbSS genotype being most common (>70%) and most severe, followed by HbSC genotype. SCD affects predominantly people of African and Afro-Caribbean descent and evidence demonstrates inequalities in healthcare outcomes and access for people with SCD.^2^ In the UK, an all-party parliamentary group inquiry reported low awareness of SCD and its complications among healthcare professionals and underinvestment in sickle cell research.^3^

Proliferative sickle cell retinopathy (SCR) is the most common ophthalmic manifestation of SCD and can result in significant sight loss, although the prevalence in the UK is not currently known. Decade-old retrospective and cohort studies from other countries have estimated prevalence of SCR in patients with SCD at up to 48% and associated with significant sight loss in up to 12%.^4 5^ Importantly, life expectancy for SCD has increased from 14 years in 1973 to >60 years in high-income countries with improvement in management and healthcare delivery.^6^ Evidence from cohort studies and large retrospective studies demonstrates increasing incidence of SCR with age.^7 8^ As such, we could expect the prevalence of SCR and potentially associated visual impairment to be currently higher in the UK than previously reported.

Five stages of SCR were described by Goldberg in 1971.^9^ These are progressive with stages 1–3 being asymptomatic, stage 3 reflecting the presence of retinal neovascularisation in the far periphery known as seafan neovascularisation, vitreous haemorrhage (bleeding inside the eye) develops in stage 4 and rhegmatogenous and/or tractional retinal detachment in stage 5.

### Sickle cell maculopathy

In addition to the peripheral retina, SCD can also affect the macula (central retina) potentially causing visual loss, known as sickle cell maculopathy (SCM). SCM is defined as patchy areas of severe retinal thinning in the temporal macula. The exact aetiology of macular thinning remains unclear, but it may be related to the temporal macula ending along the horizontal raphe, making it a watershed zone between the vascular arcades of the retinal circulation and thus more susceptible to vascular occlusions.^10^ While high-contrast visual acuity was preserved in most patients demonstrated to have SCM in a single-centre study,^11^ irreversible scotoma, decreased retinal sensitivity and visual loss have been described in association with SCM.^12–14^ Maculopathy was not included in the Goldberg classification as its presence only became more apparent with modern imaging techniques. There is currently no treatment for vision loss due to maculopathy.

### Current interventions for SCR and maculopathy

Laser photocoagulation is currently the only intervention with high-level evidence of safety and efficacy in the management of stage 3 SCR.^15 16^ Laser photocoagulation has an indirect effect as it destroys the ischaemic retina responsible for production of vascular endothelial growth factor (VEGF) that triggers the proliferation of new blood vessels. This technique is commonly used in the treatment of diabetic retinopathy and widely available in the UK. However, it is not clear that the pathophysiology of SCR is the same as diabetic retinopathy.

For SCR, there has been a paucity of randomised controlled trials (RCT) of intervention, with only two RCTs evaluating laser treatment for stage 3, and both conducted over 30 years ago. In the first RCT of laser, Jampol *et al* 15 compared laser treatment to observation in an RCT of 167 eyes and reported 3.4% developed vitreous haemorrhage (stage 4 SCR) in the laser-treated arm versus 27.5% in the observation arm while 1% developed visual loss in the laser-treated arm versus 5% in the observation arm. Farber *et al* subsequently reported similarly positive results^16^ with a Cochrane review of the safety and efficacy data of laser treatment for stage 3 SCR concluding that laser treatment reduces the risk of visual loss at mean follow-up of 1 year and is protective against the development of vitreous haemorrhage.^17^ However, the RCTs and other case series also reported spontaneous regression of stage 3 in up to 30% of eyes.^7 17^ In addition, these RCTs evaluating laser treatment are more than 30 years old and laser technology as well as the systemic management of SCD has improved significantly in that time.

Alternatively, stage 3 and 4 SCR can be managed with intravitreal anti-VEGF therapy. Over the last 16 years, intravitreal anti-VEGF has become commonplace in ophthalmic practice with many licensed indications including age-related macular degeneration and diabetic retinopathy. As VEGF is implicated in SCR,^18^ the use of intravitreal anti-VEGF therapy is scientifically rational, and evidence of regression and resolution has been demonstrated in case series of eyes with stage 3 and 4 SCR.^19^ Additionally, angiopoietin-1 and angiopoietin-2 have also been implicated in the pathophysiology of SCR.^20^ In June 2022, the National Institute for Health and Care Excellence approved faricimab, an intravitreal bispecific monoclonal antibody that inhibits VEGF and angiopoietin-2 for the treatment of wet age-related macular degeneration and diabetic macular oedema.^21 22^ No RCTs have been conducted comparing intravitreal anti-VEGF therapy or anti-angiopoietin-2 to laser therapy or observation.

The main therapeutic intervention for stage 4 and 5 SCR is vitrectomy. Vitrectomy is a surgical intervention that involves removal of the vitreous (including blood if stage 4 SCR) and repositioning of the retina to its proper position against the back of the eye (when there is retinal detachment as in stage 5). Despite significant advances in modern surgical techniques, visual outcomes for stage 4 or 5 SCR remain poor, with final visual acuity after surgery often significantly below driving vision,^23^ so early detection of SCR remains an opportunity to prevent sight loss.

There is also the potential for systemic treatment of SCD to influence the progression of SCR and/or maculopathy. Hydroxycarbamide is the gold standard treatment for prevention of painful crises in SCD and has been proven to reduce hospital admissions in patients with SCD.^24^ Hydroxycarbamide induces fetal haemoglobin (HbF) production, thereby decreasing sickle haemoglobin (Hb) erythrocyte polymers, haemolysis and painful vaso-occlusive crisis. Rigorous investigation over the past 30 years has demonstrated the efficacy of hydroxycarbamide in reducing disease complications, healthcare utilisation and costs for patients with SCD. However, compliance rates are well established to be low and linked to a variety of reasons including fear of side effects, stigma and low health literacy.^25^ Although there are no prospective studies that evaluate the efficacy of hydroxycarbamide on reducing the incidence of SCR, large retrospective studies have highlighted the protective effect of hydroxycarbamide on SCR and maculopathy.^26 27^ These studies suggest hydroxycarbamide, by elevating levels of HbF, can significantly reduce the odds of developing SCR and reduce the progression of sickle maculopathy. There is also emerging evidence that the severity of SCR and maculopathy correlates with severity of systemic anaemia associated with SCD.^28 29^ As such, retinal screening may improve compliance with hydroxycarbamide if evidence confirming protective retinal effects are corroborated. Retinal screening may also highlight the need for escalations of management to the haematologist.

### Retinal imaging for SCR and maculopathy

Retinal imaging has undergone revolutionary advancement in the past 20 years. Ultra-widefield fundus photography detects SCR more reliably than traditional eye examination or imaging.^30^ The widespread availability and use of optical coherence tomography (OCT) (and OCT angiography (OCTA) in some centres), which are rapid, non-invasive imaging tools that produce detailed images of the macular and associated vascular plexuses, means we can now detect damage to the macular otherwise undetected using traditional methods. These tools are now used routinely and extensively in the management of various high-volume eye diseases across the country including age-related macular degeneration, diabetic eye disease and patients with visual symptoms from SCR or maculopathy and are likely to be acceptable to all patients with SCR. The combination of ultra-widefield colour, OCT and OCTA will provide the best method for detecting vision-threatening retinal changes in SCD and detecting change over time.

### Clinical study rationale

There is no reliable estimate of the burden of sight loss due to SCR and maculopathy in the UK or the number of people with different stages of SCR and maculopathy. This lack of data makes planning configuration and delivery of appropriate services for eye health in SCD difficult and could lead to unwarranted variation.

Our study will determine the proportion of vision loss due to SCR and maculopathy in the UK. We will also determine the distribution of the stages of SCR and maculopathy and correlation with severity of SCD. SCR and maculopathy screening are easily incorporated into existing diagnostic clinical pathways for retinal diseases. However, there is a need to establish any benefit of screening and the acceptability to patients with SCD.

### Study objectives

The Sickle Eye Project includes an internal pilot phase, which is the first 4 months of the recruitment period, to assess recruitment and trial processes.

The objectives of the internal pilot are:

- To open six sites by the end of the pilot phase.
- 95% of the expected monthly recruitment is achieved by the end of the pilot phase.
- Adherence to imaging protocol in ≥90% of cases.
- 95% of recruited participants with completed National Eye Institute Visual Function Questionnaire-25 (NEI-VFQ-25) questionnaire.

### Main study primary objective

To determine the prevalence of visual impairment (best corrected visual acuity (BCVA) logarithm of the minimum angle of resolution (LogMAR) ≥0.3 in at least one eye) due to SCR and/or maculopathy in persons with SCD.

### Main study secondary objectives

- To determine the prevalence of each stage of SCR and maculopathy and correlation with: (1) number of hospital admissions with acute sickle cell episode in the past 12 months, (2) serum HbF level and (3) serum Hb level.
- To determine the impact of SCR and maculopathy on vision-related quality of life (VRQoL).
- To determine the acceptability to patients of retinal imaging and routine screening for SCR and maculopathy.

### Outcome measures

#### Internal pilot outcomes

The following progression criteria using a red/amber/green traffic light system will be used to inform whether we progress to the main trial:

##### Site opening progress

- Green: six sites are open to recruitment.
- Amber: three to five sites are open to recruitment.
- Red: two or fewer sites or more are open to recruitment.

##### Recruitment progress

- Green: recruitment of >120 at end of pilot phase.
- Amber: recruitment of <120 but ≥79 patients at end of pilot phase.
- Red: recruitment of <79 patients at end of pilot phase.

##### Fidelity check demonstrating adherence to image acquisition protocol

- Green: adherence to image acquisition protocol in ≥90% of cases.
- Amber: adherence to image acquisition protocol in ≥80%.
- Red: adherence to image acquisition protocol in <80% of cases.

##### Completion of the NEI-VFQ-25

- 100% of recruited participants completing the NEI-VFQ-25.
- Green: ≥95% of recruited participants with completed NEI-VFQ-25.
- Amber: ≥80% of recruited participants with completed NEI-VFQ-25.
- Red: <80% of recruited participants with completed NEI-VFQ-25.

If at the end of the pilot phase the thresholds are at amber or red for any of the progression decision points, the coinvestigator group, which includes a lived experience expert as coapplicant, will review and provide remedial action that would allow progression to the main study to the funder. If there are no obvious remedial actions that can be taken, the coinvestigator group will discuss stopping the study with the funder.

#### Main study primary outcome

- The prevalence of visual impairment due to SCR or maculopathy (defined as BCVA LogMAR ≥0.3 in at least one eye) in a representative sample of the UK population with SCD.

#### Secondary outcomes

- The prevalence of each stage of SCR and presence of maculopathy by age and genotype.
- The correlation of each stage of SCR and maculopathy to severity of SCD as determined by number of hospital admissions in the preceding 12 months/serum HbF/serum Hb.
- The impact of SCR and presence of maculopathy on VRQoL.
- The acceptability to patients of routine retinal imaging for SCR and maculopathy.
- The development of updated consensus definitions and a modernised classification system for SCR and maculopathy, leveraging the increased insight into retinal tissue anatomy and vascular blood flow afforded by contemporary imaging technologies.

## Methods

This is a cross-sectional, non-interventional study, with planned recruitment over 15 months. Potential participants will be introduced to the study by their haematologist, who will also provide them with the participant information sheet. Identification of potential participants in the haematology clinic is important to prevent ascertainment bias and obtain a representative sample of people with SCD. All patients with SCD are offered regular specialist follow-up by a haematologist in the UK. The National Haemoglobinopathy Registry (NHR) record for 2021 reported 12 904 people with SCD confirming that an overwhelming proportion of patients with SCD are under specialist care in the UK. Informed consent will be obtained from willing participants by the ophthalmology team either in the eye department or over the telephone. Informed consent will be obtained prior to the collection of any data. Once consented, participants will have BCVA with habitual correction and pinhole measured, a slit lamp examination of the anterior segment and fundus, spectral-domain OCT (SD-OCT), OCTA and ultra-widefield fundus photography performed in the eye department. Subsequently, the participant will have the NEI-VFQ-25 questionnaire and the acceptability of retinal screening questionnaire administered.

The NEI-VFQ-25 questionnaire can be administered over the telephone either at the time of consent or after the visit to the eye department. The retinal screening questionnaire is to be administered to the participant once their retinal imaging studies have been performed.

If the NEI-VFQ-25 is completed over the telephone, this must be done ±28 days of the eye clinic visit for examination and retinal images. The participant is subsequently asked if there have been any acute changes to their visual function.

The retinal images acquired from study participants will be uploaded to the Moorfields Reading Centre where they will be double graded with adjudication by a senior retinal specialist.

The research team will also complete an electronic case report form recording basic demographic history such as: year of birth, sex, sickle genotype, number of hospital admissions with acute sickle crisis, medical history, current medications and interventions for SCD, history of stage 4 or 5 SCR, ocular comorbidities, previous ocular interventions and most recent serum Hb and serum haemoglobin F (HbF) within 3 months of study entry.

The planned study start date for participant recruitment is 14 November 2023 and planned recruitment end date is 28 February 2025.

### Patient and public involvement

Our patient and public involvement and engagement (PPIE) advisors, consisting of 16 people with lived experience of SCD, have been involved in defining the research question and methodology for this study. The study management group also includes a coapplicant with lived experience of SCD who has been involved in the strategic management of the study, including design of public engagement meetings, social media engagement with the public, and during the recruitment phase will be involved in strategic decision-making for the effective delivery of the study. Our PPIE advisors informed us of the general lack of awareness of the potential effects of SCD on vision. They described the difficulties associated with vision loss including the increased vulnerability associated with loss of a driving licence in the context of living with SCD, days off work associated with vitreous haemorrhage impacting their financial well-being and livelihoods and difficulty engaging in vision-related tasks at work and university. One of the members of our PPIE group told us they would have been compliant with their systemic medication if they knew it could help preserve their sight, and the others agreed this could be a motivating factor for many people with SCD. This group will be involved in any changes to recruitment strategy and drafting the dissemination plan.

### Participants and recruitment

Haematology teams in participating sites will inform consecutive patients ≥16 years with SCD who meet the study criteria about the study, providing them with the participant information sheet. They will refer interested participants to the ophthalmology unit for study participation. Participating trusts have been selected as they already offer or are willing to offer routine eye screening for patients with SCD as part of standard of care and confirmed to have ultra-widefield fundus imaging and SD-OCT. We have used the 2021 NHR report to identify centres treating patients with SCD in England. Eligible centres had to meet the following criteria:

- At least 60 patients with SCD on the registry.
- Ultra-widefield fundus photography.
- SD-OCT imaging.
- A routine screening programme for SCR in place or willing to institute one.
- Capacity and willingness to participate in the study.

600 individuals who have been diagnosed at any point with SCD will be identified from up to 16 geographically dispersed participating National Health Service (NHS) trusts across the UK.

An invitation letter informing potential participants due to attend a sickle cell clinic can be sent out with appointment letters so that they are aware of the study before attending the clinic.

Interested participants will attend the eye clinic where they will be required to provide written informed consent and research activities will occur. For those who do not speak English or would benefit from translation services, this will be funded by the study. Informed consent can also be obtained over the telephone. Potentially eligible participants who have previously given consent to contact for any research can be approached by the research team by telephone and if willing to consider participating, the patient information sheet can be posted to them. There will be an opportunity to discuss the study before consent is obtained in the eye clinic or by telephone, and it will be made clear that the individual is free to withdraw from the study at any point.

### Eligibility criteria

#### Inclusion criteria

- Willingness to participate.
- Ability to provide informed consent.
- Age 16 years or older.
- Diagnosis of SCD of any genotype.

#### Exclusion criteria

- Inability to consent.
- Poor image quality.
- Age <16 years.
- Sickle cell trait only.

### Study activities

A summary of the schedule of activities is listed in table 1.

**Table 1:** Schedule of activities, study personnel responsible and study timeline

Study activity	Personnel	Baseline	±28 days
Eligibility criteria check	Haematology team	x	
Patient information sheet	Haematology team	x	
Informed consent*	Ophthalmology team	x (Can be completed by telephone)	
BCVA with habitual correction and pinhole	Ophthalmology team	x	
Slit lamp biomicroscopy and dilated funduscopy	Ophthalmology team	x	
OCT	Ophthalmology team	x	
OCTA	Ophthalmology team	x	
Ultra-widefield fundus photography	Ophthalmology team	x	
NEI-VFQ-25*	Ophthalmology team	x (Can be completed by telephone)	x
Acceptability questionnaire*	Ophthalmology team	x (Can be completed by telephone after retinal imaging and within 14 days)	x
Clinical assessment of images	Ophthalmology	x	
Completion of case report form	Ophthalmology/haematology team	x	x
Transfer of images	Ophthalmology team	x	x
Grading of images	Moorfields Reading Centre		Performed in batches throughout the study period

*Study activities that can be completed over the phone.

BCVA, best corrected visual acuity; NEI-VFQ-25, National Eye Institute Visual Function Questionnaire-25; OCT, optical coherence tomography; OCTA, OCT angiography.

#### Theoretical framework of acceptability-based acceptability questionnaire

The theoretical framework of acceptability (TFA) was developed in response to recommendations that acceptability should be assessed in the design, evaluation and implementation phases of healthcare interventions.^31^ The TFA consists of seven component constructs (affective attitude, burden, ethicality, intervention coherence, opportunity costs, perceived effectiveness and self-efficacy) that can help identify characteristics of interventions that may be improved. Using this robust framework, a validated generic TFA-based questionnaire^32^ was developed as an adaptable tool to measure acceptability across various healthcare interventions.

#### Prevalidating the questionnaire for acceptability of routine retinal screening in persons with SCD

We have adapted the generic TFA questionnaire with insights from our PPIE group pilot, expert clinical opinion and literature review. The first draft of the adapted TFA questionnaire was further reviewed by 10 members of our PPIE group. This feedback was incorporated and shared with a further group of 10 patients attending retinal screening clinics for SCD at two eye units in the UK to check comprehensibility and comprehensiveness. The final version of the questionnaire incorporates this input from patients and medical experts managing patients with SCD.

### Other study activities

#### BCVA with habitual correction and pinhole

This is a measure of the primary outcome. BCVA will be assessed using the Early Treatment of Diabetic Retinopathy Study vision chart in a 4 m lane under routine clinic conditions. Participants will be invited to bring their most up-to-date correction to the study visit. BCVA will be recorded with their habitual correction and pinhole in LogMAR. Refraction by an optometrist is not required.

#### Slit lamp biomicroscopy and dilated funduscopy

Slit lamp biomicroscopy allows the ophthalmologist to microscopically examine the eye for any abnormalities or problems. Dilated funduscopy allows better evaluation of the lens and vitreous. This will be performed to document other ocular causes of visual impairment such as corneal scars, cataracts and vitreous haemorrhage.

#### Spectral-domain OCT

SD-OCT is the current reference standard for diagnosing and monitoring progression of various macular diseases. In SCM, there is evidence of retinal thinning, with the temporal macular being most commonly affected.

#### OCT angiography

OCTA provides depth-resolved visualisation of the superficial and deep capillary plexuses. By allowing depth-resolved visualisation of the macular vascular network with high resolution, OCT- angiography (OCTA) recently detected much more macular vascular alterations than previously detected using just OCT and provided additional features, including enlargement and irregularities of the foveal avascular zone, hairpin venular loops and areas of flow loss responsible for irreversible macular thinning.^33^

#### Ultra-widefield fundus photography

Ultra-widefield fundus photography captures an image covering more than 80% of the retina. It allows the observer to assess the retina, associated blood vessels and optic nerve. This is particularly of benefit in SCR, where the initial stages of proliferative retinopathy occur in the far periphery and can be missed if the periphery is not carefully assessed. All units included in this study will be using either the Optos or the Clarus. While there is evidence that undilated ultra-widefield fundus photography can be of utility in screening for SCR with a specificity of 100% compared with dilated funduscopy examination in one study, the sensitivity was 69.2%.^34^ Therefore, for the purpose of this study, ultra-widefield fundus photography will be performed with a dilated pupil.

#### National Eye Institute Visual Function Questionnaire-25

The NEI-VFQ-25 is a validated 25-item questionnaire comprising 11 subscales on different aspects of vision-related functioning (VRQoL), quality of life and one item on general health.

Due to the proliferation of diagnostic hubs in the NHS, the NEI-VFQ-25, which can take up to 15 min to complete, may limit recruitment at some sites. As such, we will monitor the impact of completing this questionnaire on recruitment through our monthly investigators meeting for the first 3 months of recruitment. Feedback from principal investigators on the impact of the NEI-VFQ-25 on recruitment will determine whether to switch to the EQ-5D with vision bolt-on.

#### EQ-5D with vision bolt-on

The 5 Level EQ-5D is a widely used preference-based health-related quality of life instrument with one question for each of five core dimensions of health: mobility, self-care, usual activities, pain/discomfort and anxiety/depression. To capture the effect of conditions that affect sensory functions or cognition that may not be adequately captured by the instrument, additional dimensions, known as ‘bolt-ons’, were developed. The vision bolt-on has demonstrated enhanced sensitivity, construct validity and responsiveness to change in populations with vision problems.^35 36^ Importantly, EQ-5D with vision bolt-on comprises six questions which can be rapidly completed by participants.

### Image acquisition protocol and transfer of data

Imaging acquisition will be performed by trained staff at each recruitment site. As this is a pragmatic study, and the imaging to be acquired is standard of care in NHS eye departments, standards for training and experience of staff performing the imaging acquisition function will be the ones applicable at each recruitment site as per local standard operating procedures.

The Moorfields Reading Centre will provide supporting material of educational and advisory character for acquisition of high-quality imaging from the Sickle Eye Project participants. It will also provide detailed, yet simple and intuitive instructions for imaging file export and direct upload to the Moorfields Grading Portal Web-based Application.

For each patient, imaging from the following three modalities will be obtained and transferred to the Moorfields Reading Centre. Imaging from both eyes will be obtained:

- OCT scan (TopCon 3D-2000, Heidelberg Spectralis or Zeiss Cirrus). Recommended parameters: macula centre 6×6 mm raster scans.
- OCTA where available (TopCon Triton, Heidelberg Spectralis OCT-2, Zeiss Angioplex). Recommended parameters: macula centre 6×6 OCTA mode.
- Widefield imaging (Optos California or Zeiss Clarus). Recommended parameters: instructions will be provided to assist imaging acquisition staff with obtaining a clear view of the fundus, including the extreme periphery through imaging acquisition at the central and four peripheral positions of gaze (up, down, right, left) and the avoidance of ‘eyelash’ artefacts that interfere with the fundus view in Optos Ultra-WideField Imaging.

The images being transferred to Moorfields Reading Centre will be link anonymised with no patient identifiers. The link key will remain with each recruiting site and will not be shared with the sponsor site.

Bidirectional communication between recruitment sites and the Moorfields Reading Centre will be possible through the interactive Moorfields Grading Portal. A granular audit log will record all study-related activities, including who, where and when each activity was performed.

## Statistics and data analysis

The primary analysis will be to calculate the prevalence of SCR/maculopathy-related vision loss together with its 95% CI to illustrate the uncertainty in the estimate. It is anticipated the prevalence will be approximately 10% based on data from Saidkasimova *et al*.^37^ With 554 subjects, the prevalence can be estimated with a 95% CI with a 5% width, for example, expected to be from 7.5% to 12.5%. The sample size (n) was chosen to satisfy this 5% width, given the CI is expected to be p±1.96*sqrt (p*(1−p)/n), where p=0.1. To obtain this number of evaluable subjects we estimate approximately 600 subjects will have to be enrolled, this allows for a dropout rate of just under 10%.

A sample size of 600 will also allow accurate estimates of the stages of SCR and maculopathy occurrence to be obtained. For example, the CIs in table 2 are based on a French retrospective cohort of 942 patients.^8^

**Table 2:** CIs from large retrospective study of sickle cell retinopathy in France

	%	n	95% CI	Width (%)
Stage 0	30	166	23 to 37	14
Stages 1–2	40	222	34 to 46	13
Stage 3	20	110	13 to 27	15
Stage 4	5	28	8 to 20	12
Stage 5	5	28	8 to 20	12
	100	554		

Other endpoints also include the calculation of the prevalence of each stage of SCR by age bracket and genotype. Sample size relevance in these circumstances can be illustrated by considering a hypothetical outcome. For example, under the null hypothesis, there would be no association between prevalence and age, and given the hypothetical distributions of stage and age (table 3), it can be shown that the estimated correlation (Spearman rank) has a greater than 3 SE difference (from zero) with 554 cases. This suggests powering is adequate for associations of this size, by the ‘rule of three’, for 85% power (Z beta=1) and 5% significance level (Z alpha=1.96).

**Table 3:** Hypothetical distribution of stage of sickle cell retinopathy and age

		Age group						Overall rate
n		3.1%	7.4%	8.2%	14.0%	16.9%	21.7%	9.7%
	**Stage/Age**	**16–25**	**26–35**	**36–45**	**46–55**	**56–65**	**66+**	
	**5**	1	5	6	6	5	2	
SCR	**4**	2	5	5	8	6	3	
	**3**	23	28	28	20	12	4	
	**1–2**	46	56	55	39	23	8	
	**0**	24	42	40	27	19	6	
		*96*	*136*	*134*	*100*	*65*	*23*	*554*
		Spearman rank −0.14 (95% CI −0.23 to −0.06), p=0.001						

SCR, sickle cell retinopathy.

### Data analysis plan

The primary analysis is to estimate the prevalence of SCD-related vision loss. In addition to the calculated value, a corresponding 95% CI will be obtained to illustrate its associated uncertainty. We will also calculate the prevalence of each stage of SCR and maculopathy by age bracket and genotype. The unit of analysis will be the eye with the worse SCR stage, or randomly chosen eye if both are of the same stage. The χ^2^ test for trend will be used if groups are ordinal. The Kruskal-Wallis test (categorical factors) or Spearman rank (ordinal factors) will be used when comparing SCR stages between groups, as above.

The association between SCD severity (as assessed by number of hospital admissions in the past year and Hb and HbF levels within 3 months) with SCR stage and presence of sickle maculopathy will be examined using Spearman rank or Pearson product correlation. Multivariable analysis, such as logistic regression, will be undertaken to assess the simultaneous effects of multiple factors on stage of SCR and presence of maculopathy. The correlation between NEI-VFQ-25 global score and acceptability with BCVA, SCR stage and presence of maculopathy will also be assessed using Spearman rank correlation.

## Ethics and dissemination

### Ethics approval

The project will meet the requirements and principles set by General Data Protection Regulations 2018 and the European Medicines Agency (Good Clinical Practice (GCP) guidelines).

No study activity will take place prior to all the approvals being in place from the relevant agencies, and permission to carry out the research has been received from the local Research and Innovation (R&I) office.

The study received Health Research Authority and Health and Care Research Wales approval (REC reference: 23/SC/0363) on 6 October 2023 and will receive local research and development (R&D) capacity and capability at participating sites. The study will be conducted in accordance with the recommendations for physicians involved in research on human subjects adopted by the 18th World Medical Assembly, Helsinki 1964 (including later revisions) and any other relevant ethical guidance. Any subsequent changes to the study conduct, design or management will be notified to original approving R&D department and any other relevant regulatory authority via a substantial amendment.

### Publication and dissemination policy

Results of this study will be submitted for publication in high-impact peer-reviewed ophthalmology and haematology journals. The manuscript will be prepared by the Chief Investigator and authorship will be determined by the Sickle Eye Project publication policy. The results will also be presented at conferences such as the Royal College of Ophthalmologists Annual Congress, the British Society of Haematologists. The study question has the potential to influence the eye care landscape for people with SCD, promote closer working relationships and collaboration between haematologists and ophthalmologists and support further research into the utilisation of retinal images in the management and assessment of treatment response in SCD. This view has been validated by the health professionals and extensive patient engagement work across the country. Methods to ensure rapid clinical impact once the results are available include: submitting the study findings to NHS England and the scientific committee in charge of generating guidelines by the Royal College of Ophthalmologists, production of lay information for dissemination via patient charities and organisations.

## Supplementary Material

Reviewer comments

Author's manuscript
