# Supplementary material for: Sickle Eye Project: a cross-sectional, non-interventional study of the prevalence of visual impairment due to sickle cell retinopathy and maculopathy in the UK
Source: BMJ Open. 2024 Feb 28;14(2):e082471. doi: 10.1136/bmjopen-2023-082471 (PMC10910489; doi:10.1136/bmjopen-2023-082471)
Supplement: Author's manuscript [file bmjopen-2023-082471.draft_revisions.pdf]

BMJ Open

BMJ Open is committed to open peer review. As part of this commitment we make the peer review history of every article we publish publicly available.

When an article is published we post the peer reviewers' comments and the authors' responses online. We also post the versions of the paper that were used during peer review. These are the versions that the peer review comments apply to.

The versions of the paper that follow are the versions that were submitted during the peer review process. They are not the versions of record or the final published versions. They should not be cited or distributed as the published version of this manuscript.

BMJ Open is an open access journal and the full, final, typeset and author-corrected version of record of the manuscript is available on our site with no access controls, subscription charges or pay-per-view fees (http://bmjopen.bmj.com).

If you have any questions on BMJ Open's open peer review process please email info.bmjopen@bmj.com

BMJ Open

Sickle Eye Project: Prevalence of visual impairment due to sickle cell retinopathy and maculopathy in the UK

Journal:	*BMJ Open*
Manuscript ID	bmjopen-2023-082471
Article Type:	Protocol
Date Submitted by the Author:	24-Nov-2023
Complete List of Authors:	Dinah, Christiana; London North West Healthcare NHS Trust, Ophthalmology<br>Balaskas, Konstantinos; Moorfields Eye Hospital NHS Foundation Trust, ; UCL, Institute of Ophthalmology<br>Greystoke, Brigit; Royal Victoria Infirmary<br>Awadzi, Rossby; London North West University Healthcare NHS Trust<br>Beke, Peter; Lived experience expert<br>Ahern, Roger; Independent statistician<br>Talks, S.; Newcastle Upon Tyne Hospitals NHS Foundation Trust
Keywords:	Medical retina < OPHTHALMOLOGY, Anaemia < HAEMATOLOGY, Health Services

SCHOLARONE™
Manuscripts

Sickle Eye Project: Prevalence of visual impairment due to sickle cell retinopathy and maculopathy in the UK

Authors: Christiana Dinah[1,2]§, Konstantinos Balaskas[3], Brigit Greystokes[4], Rossby Awadzi[1], Peter Beke[5], Roger A'hern[6], James Talks[4]

§ **Corresponding author address:**

Christiana Dinah
Ophthalmology Department
London Northwest University Healthcare NHS Trust
Central Middlesex Hospital
London, United Kingdom
Email: christiana.dinah@nhs.net

Author affiliations:

[1] London North West University Healthcare NHS Trust
[2]Brain Sciences Department, Imperial College London
[3] NIHR Moorfields Biomedical Research Centre, Moorfields Eye Hospital NHS Foundation Trust, London, UK
[4] Royal Victoria Infirmary, Newcastle
[5]Lived experience expert
[6] Independent Statistician

Keywords: medical retina, anaemia, health services

Word count: 4899

For peer review only

ABSTRACT

Introduction

Sickle-cell disease (SCD) is one of the most common genetic disorders in the UK, with over 15000 people affected. Proliferative sickle cell retinopathy (SCR) is a well-described complication of SCD and can result in significant sight loss, although the prevalence in the UK is not currently known. There are currently no national screening guidelines for sickle cell retinopathy, with wide variations in the management of the condition across the UK.

Methods and analysis

The Sickle Eye Project is an epidemiological, cross-sectional, non-interventional study to determine the prevalence of visual impairment due to SCR and/or maculopathy in the UK. Haematologists in at least 16 geographically dispersed hospitals in the UK linked to participating eye clinics will offer study participation to consecutive patients meeting the inclusion criteria attending the sickle cell clinic. The following study procedures will be performed: a) best corrected visual acuity with habitual correction and pinhole b) dilated slit lamp biomicroscopy and funduscopy c) optical coherence tomography (OCT) d) OCT-angiography where available e) ultrawide fundus photography f) NEI-VFQ 25 g) acceptability of retinal screening questionnaire. The primary outcome is the proportion of people with sickle cell disease with visual impairment defined as LogMAR ≥0.3 in at least one eye. Secondary outcomes include the prevalence of each stage of sickle cell retinopathy and presence of maculopathy by age and genotype; correlation of stage of sickle cell retinopathy and maculopathy to severity of SCD, the impact of sickle cell retinopathy and presence of maculopathy on vision-related quality of life and the acceptability to patients of routine retinal imaging for sickle cell retinopathy and maculopathy.

Ethics and dissemination

Ethical approval was obtained from the **South Central – Oxford A Research Ethics Committee (REC** 23/SC/0363). Findings will be reported through academic journals in ophthalmology and haematology.

For peer review only

For peer review only - http://bmjopen.bmj.com/site/about/guidelines.xhtml

ARTICLE SUMMARY

Strengths and limitations of this study

- The Sickle Eye Project is an epidemiological, cross-sectional, non-interventional multi-centre study that will determine the prevalence of visual impairment due to sickle cell retinopathy and/or maculopathy in the United Kingdom
- To our knowledge, it is the first multi-center study of ocular complications of sickle cell disease in the UK and the first to utilize ultrawide field fundus photography, optical coherence tomography and optical coherence tomography-angiography to phenotype retinal complications of sickle cell disease.
- The study will assess the correlation between surrogates of severity of sickle cell disease and retinal imaging biomarkers
- The study incorporates double-grading by expert graders and retinal specialists at Moorfields Reading Centre
- One limitation is that the prevalence data and the imaging findings pertain to people with sickle cell disease in the United Kingdom and may not be directly generalizable toother health systems, especially Africa and Asia, which are the continents with the highest prevalence of sickle cell disease.

BACKGROUND

Proliferative sickle cell retinopathy

Sickle-cell disease (SCD) is one of the most common genetic disorders in the UK, with approximately 15000 people affected (1). It is a debilitating, multisystem disease, associated with episodes of acute illness, progressive organ damage and reduced life expectancy. There are 13 genetic variants of SCD, with HbSS genotype being most common (>70%) and most severe, followed by HbSC genotype. SCD affects predominantly people of African and Afro-Caribbean descent and evidence demonstrates inequalities in healthcare outcomes and access for people with SCD (2). In the UK, an all-party parliamentary group inquiry reported low awareness of SCD and its complications among healthcare professionals and underinvestment in sickle cell research (3).

Proliferative sickle cell retinopathy (SCR) is the most common ophthalmic manifestation of SCD and can result in significant sight loss, although the prevalence in the UK is not currently known. Decades old retrospective

For peer review only - http://bmjopen.bmj.com/site/about/guidelines.xhtml

and cohort studies from other countries have estimated prevalence of SCR in SCD patients at up to 48% and associated with significant sight loss in up to 12% (4,5). Importantly, life expectancy for SCD has increased from 14 years in 1973 to >60 years in high-income countries with improvement in management and healthcare delivery (6). Evidence from cohort studies and large retrospective studies demonstrate increasing incidence of SCR with age (7,8). As such, we could expect the prevalence of SCR and potentially associated visual impairment, to be currently higher in the UK than previously reported.

Five stages of SCR were described by Goldberg in 1971 (9). These are progressive with stages 1-3 being asymptomatic, stage 3 reflecting the presence of retinal neovascularization in the far periphery known as seafan neovascularization, vitreous haemorrhage (bleeding inside the eye) develops in stage 4 and rhegmatogenous and/or tractional retinal detachment in stage 5.

Sickle cell maculopathy

In addition to the peripheral retina, SCD can also affect the macula (central retina) potentially causing visual loss, known as sickle cell maculopathy. Sickle cell maculopathy (SCM) is defined as patchy areas of severe retinal thinning in the temporal macula. The exact aetiology of macular thinning remains unclear, but it may be related to the temporal macula ending along the horizontal raphe, making it a watershed zone between the vascular arcades of the retinal circulation and thus more susceptible to vascular occlusions(10). Whilst high-contrast visual acuity was preserved in most patients demonstrated to have SCM in a single center study (11), irreversible scotoma, decreased retinal sensitivity and visual loss has been described in association with SCM (12,13,14). Maculopathy was not included in the Goldberg classification as its presence only became more apparent with modern imaging techniques. There is currently no treatment for vision loss due to maculopathy.

Current interventions for SCR and maculopathy

Laser photocoagulation is currently the only intervention with high-level evidence of safety and efficacy in the management of stage 3 SCR (15,16). Laser photocoagulation has an indirect effect as it destroys the ischaemic retina responsible for production of vascular endothelial growth factor (VEGF) that triggers the proliferation of new blood vessels. This technique is commonly used in the treatment of diabetic retinopathy and widely available in the UK However, it is not clear that the pathophysiology of sickle cell retinopathy is the same as diabetic retinopathy.

For SCR, there has been a paucity of randomized controlled trials (RCT) of intervention, with only 2 RCTs evaluating laser treatment for stage 3, and both conducted over 30 years ago. In the first randomized controlled trial of laser, Jampol et al (15) compared laser treatment to observation in a randomized controlled trial of 167

For peer review only - http://bmjopen.bmj.com/site/about/guidelines.xhtml

eyes and reported 3.4% developed vitreous haemorrhage (stage 4 SCR) in the laser-treated arm versus 27.5% in the observation arm whilst 1% developed visual loss in the laser-treated arm versus 5% in the observation arm. Faber et al subsequently reported similarly positive results (16) with a Cochrane review of the safety and efficacy data of laser treatment for stage 3 SCR concluding that laser treatment reduces the risk of visual loss at mean follow-up of 1 year and is protective against the development of vitreous haemorrhage (17). However, the RCTs and other case series also reported spontaneous regression of stage 3 in up to 30% of eyes (7, 17). In addition, these RCTs evaluating laser treatment are more than 30 years old and laser technology as well as the systemic management of SCD has improved significantly in that time.

Alternatively, stage 3 and 4 SCR can be managed with intravitreal anti-vascular endothelial growth factor (anti-VEGF) therapy. Over the last 16 years, intravitreal anti-VEGF have become commonplace in ophthalmic practice with many licensed indications including age-related macular degeneration and diabetic retinopathy. As VEGF is implicated in SCR (18), the use of intravitreal anti-VEGF therapy is scientifically rational, and evidence of regression and resolution has been demonstrated in case series of eyes with stage 3 and 4 SCR (19). Additionally, angiopoietin-1 and angiopoietin-2 have also been implicated in the pathophysiology of SCR (20). In June 2022, NICE approved Faricimab, an intravitreal bi-specific monoclonal antibody that inhibits VEGF and angiopoietin-2 for the treatment of wet-age related macular degeneration and diabetic macular oedema (21,22). No RCTs have been conducted comparing intravitreal anti-VEGF therapy or anti-angiopoietin-2 to laser therapy or observation.

The main therapeutic intervention for stage 4 and 5 SCR is vitrectomy. Vitrectomy is a surgical intervention that involves removal of the vitreous (including blood if stage 4 SCR) and repositioning of the retina to its proper position against the back of the eye (when there is retinal detachment as in stage 5). Despite significant advances in modern surgical techniques, visual outcomes for stage 4 or 5 SCR remain poor, with final visual acuity post-surgery often significantly below driving vision (23), so early detection of SCR remains an opportunity to prevent sight loss.

There is also the potential for systemic treatment of SCD to influence the progression of SCR and/or maculopathy. Hydroxycarbamide is the gold standard treatment for prevention of painful crises in SCD and has been proven to reduce hospital admissions in patients with SCD (24). Hydroxycarbamide induces fetal haemoglobin production (HbF) thereby decreasing sickle haemoglobin erythrocyte polymers, haemolysis, and painful vaso-occlusive crisis. Rigorous investigation over the past 30 years has demonstrated the efficacy of hydroxycarbamide in reducing disease complications, health care utilization, and costs for patients with SCD. However, compliance rates are well established to be low and linked to a variety of reasons including fear of side effects, stigma and low health literacy (25). Although there are no prospective studies that evaluate the

For peer review only - http://bmjopen.bmj.com/site/about/guidelines.xhtml

efficacy of hydroxycarbamide on reducing the incidence of SCR, large retrospective studies have highlighted the protective effect of hydroxycarbamide on SCR and maculopathy (26,27). These studies suggest hydroxycarbamide, by elevating levels of HbF, can significantly reduce the odds of developing SCR and reduce the progression of sickle maculopathy. There is also emerging evidence that the severity of SCR and maculopathy correlates with severity of systemic anaemia associated with SCD (28,29). As such, retinal screening may improve compliance with hydroxycarbamide if evidence confirming protective retinal effects are corroborated. Retinal screening may also highlight the need for escalations of management to the haematologist.

Retinal imaging for SCR and maculopathy

Retinal imaging has undergone revolutionary advancement in the past 20 years. Ultrawide field fundus photography detects SCR more reliably than traditional eye examination or imaging (30). The widespread availability and use of optical coherence tomography (OCT) (and OCT-angiography in some centers), which are rapid, non-invasive imaging tools that produce detailed images of the macular and associated vascular plexuses means we can now detect damage to the macular otherwise undetected using traditional methods. These tools are now used routinely and extensively in the management of various high-volume eye diseases across the country including age-related macular degeneration, diabetic eye disease and patients with visual symptoms from SCR or maculopathy and are likely to be acceptable to all patients with SCR. The combination of ultra-widefield colour, OCT and OCTA will provide the best method for detecting vision-threatening retinal changes in sickle cell disease and detecting change over time.

Clinical study rationale

There is no reliable estimate of the burden of sight loss due to SCR and maculopathy in the UK or the number of people with different stages of SCR and maculopathy. This lack of data makes planning configuration and delivery of appropriate services for eye health in SCD difficult and could lead to unwarranted variation

Our study will determine the proportion of vision loss due to SCR and maculopathy in the UK. We will also determine the distribution of the stages of SCR and maculopathy and correlation with severity of SCD. SCR and maculopathy screening are easily incorporated into existing diagnostic clinic pathways for retinal diseases. However, there is a need to establish any benefit of screening and the acceptability to patients with SCD.

For peer review only - http://bmjopen.bmj.com/site/about/guidelines.xhtml

Study Objectives

The Sickle Eye Project includes an internal pilot phase, which is the first 4 months of the recruitment period, to assess recruitment and trial processes.

The objectives of the internal pilot are:

1. To open 6 sites by the end of the pilot phase
2. 95% of the expected monthly recruitment is achieved by the end of the pilot phase
3. Adherence to imaging protocol in ≥90% of cases
4. 95% of recruited participants with completed NEI VFQ 25 questionnaire

Main study primary objective: To determine the prevalence of visual impairment (best corrected visual acuity ≥LogMAR 0.3 in at least one eye) due to SCR and/or maculopathy in persons with SCD

Main study secondary objectives:

1. To determine the prevalence of each stage of SCR and maculopathy and correlation with:

i) number of hospital admissions with acute sickle cell episode in the past 12 months, ii) serum HbF level iii) serum Hb level

2. To determine the impact of SCR and maculopathy on vision-related quality of life.

3.To determine the acceptability to patients of retinal imaging and routine screening for SCR and maculopathy.

Outcome measures

Internal pilot outcomes

For peer review only - http://bmjopen.bmj.com/site/about/guidelines.xhtml

The following progression criteria using a red/amber/green traffic light system will be used to inform whether we progress to the main trial:

Site opening progress

Green: 6 sites are open to recruitment

Amber: 3 -5 sites are open to recruitment

Red: 2 or fewer sites or more are open to recruitment

Recruitment progress

Green: Recruitment of >120 at end of pilot phase

Amber: Recruitment of ≤ 120 but ≥ 79 patients at end of pilot phase

Red: Recruitment of <79 patients at end of pilot phase.

Fidelity check demonstrating adherence to image acquisition protocol

Green: Adherence to image acquisition protocol in ≥90% of cases.

Amber: Adherence to image acquisition protocol in ≥80%

Red: Adherence to image acquisition protocol in <80% of cases

Completion of the NEI VFQ-25

100% of recruited participants completing the NEI VFQ-25

Green: ≥95% of recruited participants with completed NEI VFQ-25

Amber: ≥80% of recruited participants with completed NEI VFQ-25

Red: <80% of recruited participants with completed NEI VFQ-25

If at the end of the pilot phase the thresholds are at amber or red for any of the progression decision points, the Co-Investigator Group, which includes a lived experience expert as co-applicant will review and provide

For peer review only - http://bmjopen.bmj.com/site/about/guidelines.xhtml

remedial action that would allow progression to the main study to the Funder. If there are no obvious remedial actions that can be taken, the Co-Investigator group will discuss stopping the study with the Funder.

Main study primary outcome

1. The prevalence of visual impairment due to SCR or maculopathy (defined as best corrected visual acuity LogMAR ≥ 0.3 in at least one eye) in a representative sample of the UK population with SCD

Secondary outcomes

1. The prevalence of each stage of SCR and presence of maculopathy by age and genotype

2. The correlation of each stage of SCR and maculopathy to severity of SCD as determined by number of hospital admissions in the preceding 12 months/serum HbF/ serum Hb.

3. The impact of SCR and presence of maculopathy on vision-related quality of life.

4. The acceptability to patients of routine retinal imaging for SCR and maculopathy

5. The development of updated consensus definitions and a modernized classification system for SCR and maculopathy, leveraging the increased insight into retinal tissue anatomy and vascular blood flow afforded by contemporary imaging technologies

METHODS

This is a cross-sectional, non-interventional study, with planned recruitment over 15 months. Potential participants will be introduced to the study by their haematologist, who will also provide them with the participant information sheet. Identification of potential participants in the haematology clinic is important to prevent ascertainment bias and obtain a representative sample of people with SCD. All patients with SCD are offered regular specialist follow-up by a haematologist in the UK. The national haemoglobinopathy registry record for 2021 reported 12904 people with SCD confirming that an overwhelming proportion of SCD patients are under specialist care in the UK. Informed consent will be obtained from willing participants by the ophthalmology team either in the eye department or over the telephone. Informed consent will be obtained prior to the collection of any data. Once consented, participants will have best corrected visual acuity (BCVA) with habitual correction and pinhole measured, a slit lamp examination of the anterior segment and fundus, spectral-domain optical coherence tomography (SD-OCT), optical coherence tomography-angiography (OCT-A) and ultra-widefield

For peer review only - http://bmjopen.bmj.com/site/about/guidelines.xhtml

fundus photography performed in the eye department. Subsequently, the participant will have the NEI VFQ-25 questionnaire and the acceptability of retinal screening questionnaire administered.

The NEI VFQ-25 questionnaire can be administered over the telephone either at the time of consent or after the visit to the eye department. The retinal screening questionnaire is to be administered to the participant once their retinal imaging studies have been performed.

If the NEI VFQ-25 is completed over the telephone, this must be done ±28 days of the eye clinic visit for examination and retinal images. The participant is then asked if there have been any acute changes to their visual function.

The retinal images acquired from study participants will be uploaded to the Moorfields Reading Centre where they will be double graded with adjudication by a senior retinal specialist.

The research team will also complete an electronic case report form recording basic demographic history such as: Year of birth, Sex, Sickle genotype, Number of hospital admissions with acute sickle crisis, medical history, current medications and interventions for SCD, previous history of stage 4 or 5 sickle cell retinopathy, ocular comorbidities, previous ocular interventions and most recent serum haemoglobin (Hb) and serum haemoglobin F (HbF) within 3 months of study entry.

Patient and Public Involvement statement

Our patient and public involvement and engagement (PPIE) advisors, consisting of 16 people with lived experience of SCD have been involved in defining the research question and methodology for this study. The study management group also includes a co-applicant with lived experience of sickle cell disease who has been involved in the strategic management of the study including design of public engagement meetings, social media engagement with the public and during the recruitment phase will be involved in strategic decision-making for the effective delivery of the study. Our PPIE advisors informed us of the general lack of awareness of the potential effects of SCD on vision. They described the difficulties associated with vision loss including the increased vulnerability associated with loss of a driving license in the context of living with SCD, days off work associated with vitreous haemorrhage impacting their financial well-being and livelihoods and difficulty engaging in vision-related tasks at work and university. One of the members of our PPIE group told us they would have been compliant with their systemic medication if they knew it could help preserve their sight and the others agreed this could be a motivating factor for many people with SCD. This group will be involved in any changes to recruitment strategy and drafting the dissemination plan.

For peer review only

Participants and recruitment

Haematology teams in participating sites will inform consecutive patients with SCD ≥ 16 years who meet the study criteria about the study and refer to the ophthalmology unit for study participation. Participating Trusts have been selected as they already offer or are willing to offer routine eye screening for SCD patients as part of standard of care and confirmed to have ultra-widefield fundus imaging and spectral domain OCT. We have used the 2021 national haemoglobinopathy registry (NHR) report to identify centers treating patients with SCD in England. Eligible centers had to meet the following criteria:

1. At least 60 patients with SCD on the registry
2. Ultra-wide field fundus photography
3. Spectral domain OCT imaging
4. A routine screening programme for SCR in place or willing to institute one
5. Capacity and willingness to participate in the study

600 individuals who have been diagnosed at any point with SCD will be identified from up to 16 geographically dispersed participating NHS Trusts across the UK.

An invitation letter informing potential participants due to attend a sickle cell clinic can be sent out with appointment letters so that they are aware of the study before attending the clinic.

Interested participants will attend the eye clinic where they will be consented and research activities will occur. For those who do not speak English or would benefit from translation services, this will be funded by the study. Informed consent can also be obtained over the telephone. Potentially eligible participants who have previously given consent to contact for any research can be approached by the research team by telephone and if willing to consider participating, the patient information sheet (PIS) can be posted to them. There will be an opportunity to discuss the study before consent is obtained in the eye clinic or by telephone, and it will be made clear that the individual is free to withdraw from the study at any point.

Eligibility criteria:

Inclusion criteria

- Willingness to participate
- Ability to provide informed consent

For peer review only - http://bmjopen.bmj.com/site/about/guidelines.xhtml

- Age 16 years or older
- Diagnosis of SCD of any genotype

Exclusion criteria

- Inability to consent
- Poor image quality
- Age <16 years
- Sickle cell trait only

Study activities (Summarized in Table 1)

Theoretical framework of acceptability (TFA)-based acceptability questionnaire

The theoretical framework of acceptability (TFA) was developed in response to recommendations that acceptability should be assessed in the design, evaluation and implementation phases of healthcare interventions (31). The TFA consists of seven component constructs (affective attitude, burden, ethicality, intervention coherence, opportunity costs, perceived effectiveness, and self-efficacy) that can help to identify characteristics of interventions that may be improved. Using this robust framework, a validated generic TFA-based questionnaire (32) was developed as an adaptable tool to measure acceptability across various healthcare interventions.

Pre-validating the questionnaire for acceptability of routine retinal screening in persons with SCD

We have adapted the generic TFA questionnaire with insights from our PPI group pilot, expert clinical opinion and literature review. The first draft of the adapted TFA questionnaire has been discussed with 10 members of our PPIE group. This feedback was incorporated and shared with a further group of 10 patients attending retinal screening clinics for SCD at 2 eye units in the UK to check comprehensibility and comprehensiveness. The final version of the questionnaire incorporates this input from patients and medical experts managing patients with SCD.

For peer review only - http://bmjopen.bmj.com/site/about/guidelines.xhtml

Other study activities

Best corrected visual acuity (BCVA) with habitual correction and pinhole

This is a measure of the primary outcome. BCVA will be assessed using the Early Treatment of Diabetic Retinopathy Study vision chart in a 4-meter lane under routine clinic conditions. Participants will be invited to bring their most up to date correction to the study visit. BCVA will be recorded with their habitual correction and pinhole in LogMAR. **Refraction by an optometrist is not required**

Slit lamp biomicroscopy and dilated funduscopy

Slit lamp biomicroscopy allows the ophthalmologist to microscopically examine the eye for any abnormalities or problems. Dilated funduscopy allows better evaluation of the lens and vitreous. This will be performed to document other ocular causes of visual impairment such as corneal scars, cataracts and vitreous haemorrhage.

Spectral domain OCT

Spectral domain OCT (SD-OCT) is the current reference standard for diagnosing and monitoring progression of various macular diseases. In sickle cell maculopathy, there is evidence of retinal thinning, with the temporal macular being most commonly affected.

OCT angiography

OCT-Angiography (OCT-A) provides depth-resolved visualization of the superficial and deep capillary plexuses. By allowing depth-resolved visualization of the macular vascular network with high resolution, optical coherence tomography angiography (OCTA) recently detected much more macular vascular alterations than previously detected using just OCT and provided additional features, including enlargement and irregularities of the foveal avascular zone (FAZ), hairpin venular loops, and areas of flow loss responsible for irreversible macular thinning (33).

Ultra-widefield fundus photography

Ultra-widefield fundus photography captures an image covering more than 80% of the retina. It allows the observer to assess the retina, associated blood vessels and optic nerve. This is particularly of benefit in sickle cell retinopathy, where the initial stages of proliferative retinopathy occur in the far periphery and can be missed if the periphery is not carefully assessed. All units included in this study will be using either the Optos or the Clarus. Whilst there is evidence that un-dilated ultrawide field fundus photography can be of utility in screening for sickle cell retinopathy with a specificity of 100% compared to dilated funduscopy examination in one study,

For peer review only - http://bmjopen.bmj.com/site/about/guidelines.xhtml

the sensitivity was 69.2% (34). Therefore, for the purpose of this study, ultra-widefield fundus photography will be performed with a dilated pupil.

NEI-VFQ 25

The NEI-VFQ 25 is a validated 25 item questionnaire comprising of 11 subscales on different aspects of vision-related functioning (VRQoL), quality of life and 1 item on general health.

Due to the proliferation of diagnostic hubs in the NHS, the NEI-VFQ-25, which can take up to 15 minutes to complete may limit recruitment at some sites. As such, we will monitor the impact of completing this questionnaire on recruitment through our monthly investigators meeting for the first 3 months of recruitment. Feedback from Principal Investigators on the impact of the NEI-VFQ 25 on recruitment will determine whether to switch to the EQ-5D with vision bolt-on.

EQ-5D with vision bolt-on

The 5 level EQ-5D is a widely used preference-based health related quality of life instrument with one question for each of 5 core dimensions of health: mobility, self-care, usual activities, pain/discomfort and anxiety/depression. To capture the effect of conditions that affect sensory functions or cognition that may not be adequately captured by the instrument, additional dimensions, known as 'bolt-ons' were developed. The vision bolt-on has demonstrated enhanced sensitivity, construct validity and responsiveness to change in populations with vision problems (35,36). Importantly, EQ-5D with vision bolt-on comprises of 6 questions which can be rapidly completed by participants.

For peer review only

For peer review only - http://bmjopen.bmj.com/site/about/guidelines.xhtml

Study activity	Personnel	Baseline	±28 days
**Eligibility criteria check**	Haematology team	x	
**Patient information sheet**	Haematology team	x	
**Informed consent**	Ophthalmology team	x (Can be completed by telephone)	
**BCVA with habitual correction and pinhole**	Ophthalmology team	x	
**Slit lamp biomicroscopy and dilated funduscopy**	Ophthalmology team	x	
**OCT**	Ophthalmology team	x	
**OCT-A**	Ophthalmology team	x	
**Ultrawide field fundus photography**	Ophthalmology team	x	

For peer review only - http://bmjopen.bmj.com/site/about/guidelines.xhtml

***NEI-VFQ 25**	Ophthalmology team	x (Can be completed by telephone)	x
***Acceptability Questionnaire**	Ophthalmology team	x (Can be completed by telephone after retinal imaging and within 14 days)	x
**Clinical assessment of images**	Ophthalmology	x	
**Completion of case report form**	Ophthalmology/ haematology team	x	x
**Transfer of images**	Ophthalmology team	x	x
**Grading of images**	Moorfields Reading Centre		Performed in batches throughout the study period

Table 1: Schedule of activities, study personnel responsible and study timeline

Image acquisition protocol and transfer of data

Imaging acquisition will be performed by trained staff at each recruitment site. As this is a pragmatic study, and the imaging to be acquired are standard of care in NHS eye departments, standards for training and experience of staff performing the imaging acquisition function will be the ones applicable at each recruitment site as per local Standard Operating Procedures.

For peer review only - http://bmjopen.bmj.com/site/about/guidelines.xhtml

The Moorfields Reading Centre will provide supporting material of educational and advisory character for acquisition of high-quality imaging from the Sickle Eye Project participants. It will also provide detailed, yet simple and intuitive instructions for imaging file export and direct upload to the Moorfields Grading Portal Web-based Application.

For details on the process of User activation, including Users with the Uploader role, and User Management, please see relevant provisions of the 'Data Protection and Information Security' section.

For each patient, imaging from the three following modalities will be obtained, and transferred to the Moorfields Reading Centre. Imaging from both eyes will be obtained:

- Optical Coherence Tomography scan (TopCon 3D-2000, Heidelberg Spectralis or Zeiss Cirrus).
Recommended parameters: Macula Centre 6x6 mm Raster Scans.

- Optical Coherence Tomography Angiography where available (TopCon Triton, Heidelberg Spectralis OCT-2, Zeiss Angioplex)

Recommended parameters: Macula Centre 6x6 OCT Angiography mode

- Wide Field Imaging (Optos California or Zeiss Clarus)
Recommended parameters for wide field imaging: Instructions will be provided to assist imaging acquisition staff with obtaining clear view of the Fundus, including the extreme periphery through imaging acquisition at the central and 4 peripheral positions of gaze (up, down, right, left) and the avoidance of 'eyelash' artifacts that interfere with Fundus view in Optos Ultra-Wide Field Imaging.

The images being transferred to Moorfields hospital will be link anonymized with no patient identifiers. The link key will remain with each recruiting site and will not be shared with the sponsor site.

Bi-directional communication between recruitment sites and the Moorfields Reading Centre will be possible through the interactive Moorfields Grading Portal. A granular Audit Log will record all study-related activity, including who, where and when each activity was performed.

STATISTICS AND DATA ANALYSIS

The primary analysis will be to calculate the prevalence of sickle cell retinopathy/maculopathy-related vision loss together with its 95% confidence interval (CI) to illustrate the uncertainty in the estimate. It is anticipated the

For peer review only - http://bmjopen.bmj.com/site/about/guidelines.xhtml

prevalence will be approximately 10% based on data from Saidkasimova et al (37). With 554 subjects the prevalence can be estimated with a 95% confidence interval with a 5% width e.g. expected to be from 7.5% to 12.5%. The sample size (N) was chosen to satisfy this 5% width, given the confidence interval is expected to be p +/- 1.96*sqrt(p*(1-p)/n), where p=0.1. To obtain this number of evaluable subjects we estimate approximately 600 subjects will have to be enrolled, this allows for a drop-out rate of just under 10%.

A sample size of 600 will also allow accurate estimates of the stages of SCR and maculopathy occurrence to be obtained. For example, the CIs in Table 2 are based on a French retrospective cohort of 942 patients (8) :

For peer review only

	**%**	**N**	**95%CI**	**Width**
**Stage 0**	30%	166	(23%-37%)	14%
**Stage 1-2**	40%	222	(34%-46%)	13%
**Stage 3**	20%	110	(13%-27%)	15%
**Stage 4**	5%	28	(8%-20%)	12%
**Stage 5**	5%	28	(8%-20%)	12%
	100%	554		

Table 2: Confidence intervals from large retrospective study of sickle cell retinopathy in France

Other endpoints also include the calculation of the prevalence of each stage of SCR by age bracket and genotype. Sample size relevance in these circumstances can be illustrated by considering a hypothetical outcome. For example, under the null hypothesis there would be no association between prevalence and age, and given the hypothetical distributions of stage and age below (Table 3), it can be shown that the estimated correlation (Spearman Rank) has a greater than 3 SE difference (from zero) with 554 cases. This suggests powering is adequate for associations of this size, by the 'rule of three', for 85% power (Z beta=1) and 5% sig level (Zalpha=1.96).

For peer review only - http://bmjopen.bmj.com/site/about/guidelines.xhtml

				Age Group					
**N**		*3.1%*	*7.4%*	*8.2%*	*14.0%*	*16.9%*	*21.7%*	***9.7%***	**rate overall**
	**Stage/Age**	**16-25**	**26-35**	**36-45**	**46-55**	**56-65**	**66+**		
	5	1	5	6	6	5	2		
**SCR**	4	2	5	5	8	6	3		
	3	23	28	28	20	12	4		
	1-2	46	56	55	39	23	8		
	0	24	42	40	27	19	6		
		96	*136*	*134*	*100*	*65*	*23*	554	

Spearman Rank -0.14 (95%CI:-0.23 to -0.06), p=0.001

Table 3: Hypothetical distribution of stage of sickle cell retinopathy and age

Data Analysis plan

The primary analysis is to estimate the prevalence of SCD-related vision loss. In addition to the calculated value, a corresponding 95% confidence interval will be obtained to illustrate its associated uncertainty. We will also calculate the prevalence of each stage of SCR and maculopathy by age bracket and genotype. The unit of analysis will be the eye with the worse SCR stage, or randomly chosen eye if both are of the same stage. The chi-square test for trend will be used if groups are ordinal. The Kruskal-Wallis test (categorical factors) or Spearman rank (ordinal factors) will be used when comparing SCR stages between groups, as above.

The association between SCD severity (as assessed by number of hospital admissions in past year and haemoglobin and haemoglobin F level within 3 months) with SCR stage and presence of sickle maculopathy will be examined using Spearman Rank or Pearson product correlation. Multivariable analysis, such as logistic regression, will be undertaken to assess the simultaneous effects of multiple factors on stage of SCR and

For peer review only - http://bmjopen.bmj.com/site/about/guidelines.xhtml

presence of maculopathy. The correlation between NEI VFQ-25 global score and acceptability with best corrected visual acuity, SCR stage and presence of maculopathy will also be assessed using Spearman rank correlation.

REGULATORY REVIEWS AND COMPLIANCE

Ethics approval

The project will meet the requirements and principles set by General Data Protection Regulations 2018 and the European Medicines Agency (GCP Guidelines).

No study activity will take place prior to all the approvals being in place from the relevant agencies, and permission to carry out the research has been received from the local R&I office.

The study received Health Research Authority and Health and Care Research Wales Approval, REC reference: 23/SC/0363 on the 6th of October 2023 and will receive local research and development (R&D) capacity and capability at participating sites. The study will be conducted in accordance with the recommendations for physicians involved in research on human subjects adopted by the 18th World Medical Assembly, Helsinki 1964 (incl. later revisions) and any other relevant ethical guidance. Any subsequent changes to the study conduct, design or management will be notified to original approving R&D Department and any other relevant regulatory authority via a substantial amendment.

PUBLICATION AND DISSEMINATION POLICY

Results of this study will be submitted for publication in high-impact peer reviewed Ophthalmology and haematology journals. The manuscript will be prepared by the CI and authorship will be determined by the Sickle Eye Project publication policy. The results will also be presented at conferences such as the Royal College of Ophthalmologists Annual Congress, the British Society of Haematologists. The study question has the potential to influence the eyecare landscape for people with Sickle Cell Disease, promote closer working relationships and collaboration between haematologists and ophthalmologists and support further research into the utilization of retinal images in the management and assessment of treatment response in Sickle Cell Disease. This view has been validated by the health professionals and extensive patient engagement work across the country. Methods to ensure rapid clinical impact once the results are available include: submitting the study findings to NHS England and the Scientific committee in charge of generating guidelines by the Royal College of Ophthalmologists, production of lay information for dissemination via patient charities and organizations.

For peer review only - http://bmjopen.bmj.com/site/about/guidelines.xhtml

REFERENCES

1. Dormandy E, James J, Inusa B, et al. How many people have sickle cell disease in the UK? J Public Health (Oxf) 2018;40(3):e291-e295.
2. Aljuburi G, Laverty AA, Green SA, et al. Socio-economic deprivation and risk of emergency readmission and inpatient mortality in people with sickle cell disease in England: observational study. J Public Health (Oxf) 2013;35(4):510-7.
3. Sickle Cell Society. Sickle cell disease: no one is listening. 2021. [Online]. Available at: https://www.sicklecellsociety.org./wpcontent/uploads/2021/11/No-ones-listening-final.pdf. [Accessed 11 November 2021].
4. Moriarty BJ, Acheson RW, Condon PI, et al. Patterns of visual loss in untreated sickle cell retinopathy. Eye (Lond) 1988;2(Pt 3):330-5.
5. Condon PI, Serjeant GR. Behaviour of untreated proliferative sickle retinopathy. Br J Ophthalmol 1980;64(6):404-11.
6. Gardner K, Douiri A, Drasar E, et al. Survival in adults with sickle cell disease in a high-income setting. Blood 2016;128(10):1436-8. doi: 10.1182/blood-2016-05-716910.
7. Downes SM, Hambleton IR, Chuang EL, et al. Incidence and natural history of proliferative sickle cell retinopathy: observations from a cohort study. Ophthalmology 2005;112(11):1869-75.
8. Leveziel N, Bastuji-Garin S, Lalloum F, et al. Clinical and laboratory factors associated with the severity of proliferative sickle cell retinopathy in patients with sickle cell hemoglobin C (SC) and homozygous sickle cell (SS) disease. Medicine (Baltimore) 2011;90(6):372-378.
9. Goldberg MF. Classification and pathogenesis of proliferative sickle cell retinopathy. Am J Ophthalmol 1971;71(3):649-65.
10. Stevens TS, Busse B, Lee CB, et al. Sickling hemoglobinopathies; macular and perimacular vascular abnormalities. Arch Ophthalmol 1974;92(6):455-63.
11. Sahak H, Saqalain, Lott P, et al. Sickle cell maculopathy: prevalence, associations and impact on visual acuity. Ophthalmologica 2021;244(2):159-164.
12. Lee CM, Charles HC, Smith RT, et al. Quantification of macular ischaemia in sickle cell retinopathy. Br J Ophthalmol 1987;71(7):540-545.
13. Kwok JM, Jeeva-Patel T, Margolin EA. Binasal visual field defects due to sickle cell maculopathy. J Neuroophthalmol 2022;42(1):e434-e436.
14. Chow CC, Genead MA, Anastasakis A, et al. Structural and functional correlation in sickle cell retinopathy using spectral domain optical coherence tomography and scanning laser ophthalmoscope microperimetry. Am J Ophthalmol 2011;152(4):704-711.e2.

For peer review only - http://bmjopen.bmj.com/site/about/guidelines.xhtml

15. Jampol LM, Condon P, Farber M, et al. A randomized clinical trial of feeder vessel photocoagulation of proliferative sickle cell retinopathy. I. Preliminary results. Ophthalmology 1983;90(5):540-5.
16. Farber MD, Jampol LW, Fox P, et al. A randomised clinical trial of scatter photocoagulation of proliferative sickle cell retinopathy. Arch Ophthalmol 1991;109(3):363-7.
17. Myint KT, Sahoo S, Thein AW, et al. Laser therapy for retinopathy in sickle cell disease. Cochrane Database Syst Rev 2015;(10):CD010790.
18. Cao J, Mathews MK, McLeod DS, et al. Angiogenic factors in human proliferative sickle cell retinopathy. Br J Ophthalmol 1999;83(7):838-46.
19. Cai CX, Linz MO, Scott AW. Intravitreal bevacizumab for proliferative sickle retinopathy: a case series. J Vitreoretin Dis 2018;2:32-38.
20. Mohan JS, Lip PL, Blann AD, et al. The angiopoietin/Tie-2 system in proliferative sickle cell retinopathy: relation to vascular endothelial growth factor, its soluble receptor Flt-1 and von Willebrand factor, and to the effects of laser treatment. Br J Ophthalmol 2005;89(7):815-9.
21. National Institute for Health and Care Excellence (NICE). Faricimab for treating wet age-related macular degeneration. [TA800]. 2023. Available at: https://www.nice.org.uk/guidance/ta800.
22. National Institute for Health and Care Excellence (NICE). Faricimab for treating diabetic macular oedema. [TA799]. 2023. Available at: https://www.nice.org.uk/guidance/ta799.
23. Ho J, Grabowska A, Ugarte M, et al. A comparison of 23 gauge and 20 gauge vitrectomy for proliferative sickle cell retinopathy – clinical outcomes and surgical management. Eye (Lond) 2018;32(9):1449-1454.
24. Nevitt SJ, Jones AP, Howard J. Hydroxyurea (hydroxycarbamide) for sickle cell disease. Cochrane Database Syst Rev 2017;(4):CD002202.
25. Adams-Graves P, Bronte-Jordan L. Recent treatment guidelines for managing adult patients with sickle cell disease: challenges in access to care, social issues, and adherence. Expert Rev Hematol 2016;9(6):541-52.
26. Mian UK, Tang J, Allende APM, et al. Elevated fetal haemoglobin levels are associated with decreased incidence of retinopathy in adults with sickle cell disease. Br J Haematol 2018;183(5):807-811.
27. Lim JI, Niec M, Sun J, et al. Longitudinal Assessment of Retinal Thinning in Adults With and Without Sickle Cell Retinopathy Using Spectral-Domain Optical Coherence Tomography. JAMA Ophthalmol 2021;139(3):330-337.
28. Duan XJ, Lanzkron S, Linz MO, et al. Clinical and ophthalmic factors associated with the severity of sickle cell retinopathy. Am J Ophthalmol 2019;197:105-113.
29. Fares S, Hajjar S, Romana M, et al. Sickle cell maculopathy: microstructural analysis using OCTA and identification of genetic, systemic and biological risk factors. Am J Ophthalmol 2021;224:7-17.
30. Alabduljalil T, Cheung S, Vandenhoven C, et al. Retinal ultra-wide field colour imaging versus dilated fundus examination to screen for sickle cell retinopathy. Br J Ophthalmol 2021;105:1121-6.

For peer review only
For peer review only - http://bmjopen.bmj.com/site/about/guidelines.xhtml

31. Sekhon M, Cartwright M, Francis J. Acceptability of healthcare interventions: an overview of reviews and development of a theoretical framework. BMC Health Serv Res 2017;17(1):88.
32. Sekhon M, Cartwright M, Francis JJ. Development of a theory-informed questionnaire to assess the acceptability of healthcare interventions. BMC Health Serv Res 2022;22(1):279.
33. Guerra R, Guerra C, Bastos M, et al. Sickle cell retinopathy: what we now understand using optical coherence tomography angiography. A systematic review. Blood Rev 2019;35:32-42.
34. Ahmed I, Pradeep T, Goldberg M, et al. Nonmydriatic ultra-widefield fundus photography in a hematology clinic shows utility for screening of sickle cell retinopathy. Am J Ophthalmol 2022;236:241-248.
35. Luo N, Wang X, Ang M, et al. A vision 'bolt-on' item could increase the discriminatory power of the EQ-5D index score. Value Health 2015;18(8):1037-1042.
36. Gandhi M, Ang M, Teo K, et al. A vision 'bolt-on' increases the responsiveness of EQ-5D: preliminary evidence from a study of cataract surgery. Eur J Health Econ 2020;21(4):501-511.
37. Saidkasimova S, Shalchi Z, Mahroo OA et al. Risk factors for visual impairment in patients with sickle cell disease in London. Eur J Ophthalmol. 2016;26(5):431-5.

ACKNOWLEDGMENTS

We would like to thank the sixteen members of our Sickle Cell Peer Advisory group, for their time and invaluable input into the study so far.

Author Contributions

CD, JT and KB conceptualized the project; All authors contributed to the study design. CD prepared the initial draft of the manuscript; All authors critically reviewed, edited and finalized the manuscript. All authors have read and approved the manuscript

Funding statement

This work has been supported by NIHR RFPB Grant (Ref: NIHR204961) and an unrestricted grant from Roche UK. The views expressed are those of the authors and not necessarily those of the NIHR, the department of health and social care or Roche UK.

Competing interest statement

CD reports receiving institutional research grant, consulting fees and speaker fees from Roche, KB has received consulting and speaker fees from Roche and JT has received consulting and speaker fees from Roche. The other authors have no competing interests to declare

For peer review only - http://bmjopen.bmj.com/site/about/guidelines.xhtml

For peer review only

For peer review only - http://bmjopen.bmj.com/site/about/guidelines.xhtml

STROBE Statement—Checklist of items that should be included in reports of ***cross-sectional studies***

	Item No	Recommendation	Page No
**Title and abstract**	1	(*a*) Indicate the study's design with a commonly used term in the title or the abstract	1-2
		(*b*) Provide in the abstract an informative and balanced summary of what was done and what was found	2
**Introduction**			
Background/rationale	2	Explain the scientific background and rationale for the investigation being reported	3-6
Objectives	3	State specific objectives, including any prespecified hypotheses	7
**Methods**			
Study design	4	Present key elements of study design early in the paper	9
6Setting	5	Describe the setting, locations, and relevant dates, including periods of recruitment, exposure, follow-up, and data collection	9
Participants	6	(*a*) Give the eligibility criteria, and the sources and methods of selection of participants	11
Variables	7	Clearly define all outcomes, exposures, predictors, potential confounders, and effect modifiers. Give diagnostic criteria, if applicable	13-14
Data sources/ measurement	8*	For each variable of interest, give sources of data and details of methods of assessment (measurement). Describe comparability of assessment methods if there is more than one group	*13-16*
Bias	9	Describe any efforts to address potential sources of bias	9
Study size	10	Explain how the study size was arrived at	17
Quantitative variables	11	Explain how quantitative variables were handled in the analyses. If applicable, describe which groupings were chosen and why	17-19
Statistical methods	12	(*a*) Describe all statistical methods, including those used to control for confounding	17-19
		(*b*) Describe any methods used to examine subgroups and interactions	17-19
		(*c*) Explain how missing data were addressed	
		(*d*) If applicable, describe analytical methods taking account of sampling strategy	17-19
		(*e*) Describe any sensitivity analyses	
**Results**			
Participants	13*	(a) Report numbers of individuals at each stage of study—eg numbers potentially eligible, examined for eligibility, confirmed eligible, included in the study, completing follow-up, and analysed	
		(b) Give reasons for non-participation at each stage	
		(c) Consider use of a flow diagram	
Descriptive data	14*	(a) Give characteristics of study participants (eg demographic, clinical, social) and information on exposures and potential confounders	
		(b) Indicate number of participants with missing data for each variable of interest	
Outcome data	15*	Report numbers of outcome events or summary measures	

For peer review only

Main results	16	(*a*) Give unadjusted estimates and, if applicable, confounder-adjusted estimates and their precision (eg, 95% confidence interval). Make clear which confounders were adjusted for and why they were included	
		(*b*) Report category boundaries when continuous variables were categorized	
		(*c*) If relevant, consider translating estimates of relative risk into absolute risk for a meaningful time period	
Other analyses	17	Report other analyses done—eg analyses of subgroups and interactions, and sensitivity analyses	
**Discussion**			
Key results	18	Summarise key results with reference to study objectives	
Limitations	19	Discuss limitations of the study, taking into account sources of potential bias or imprecision. Discuss both direction and magnitude of any potential bias	
Interpretation	20	Give a cautious overall interpretation of results considering objectives, limitations, multiplicity of analyses, results from similar studies, and other relevant evidence	
Generalisability	21	Discuss the generalisability (external validity) of the study results	
**Other information**			
Funding	22	Give the source of funding and the role of the funders for the present study and, if applicable, for the original study on which the present article is based	

*Give information separately for exposed and unexposed groups.

Note: An Explanation and Elaboration article discusses each checklist item and gives methodological background and published examples of transparent reporting. The STROBE checklist is best used in conjunction with this article (freely available on the Web sites of PLoS Medicine at http://www.plosmedicine.org/, Annals of Internal Medicine at http://www.annals.org/, and Epidemiology at http://www.epidem.com/). Information on the STROBE Initiative is available at www.strobe-statement.org.

For peer review only

BMJ Open

Sickle Eye Project: A cross-sectional, non-interventional study of the prevalence of visual impairment due to sickle cell retinopathy and maculopathy in the UK

Journal:	*BMJ Open*
Manuscript ID	bmjopen-2023-082471.R1
Article Type:	Protocol
Date Submitted by the Author:	18-Dec-2023
Complete List of Authors:	Dinah, Christiana; London North West Healthcare NHS Trust, Ophthalmology<br>Balaskas, Konstantinos; Moorfields Eye Hospital NHS Foundation Trust, ; UCL, Institute of Ophthalmology<br>Greystoke, Brigit; Royal Victoria Infirmary<br>Awadzi, Rossby; London North West University Healthcare NHS Trust<br>Beke, Peter; Lived experience expert<br>Ahern, Roger; Independent statistician<br>Talks, S.; Newcastle Upon Tyne Hospitals NHS Foundation Trust
**Primary Subject Heading**:	Ophthalmology
Secondary Subject Heading:	Haematology (incl blood transfusion)
Keywords:	Medical retina < OPHTHALMOLOGY, Anaemia < HAEMATOLOGY, Health Services

SCHOLARONE™
Manuscripts

For peer review only - http://bmjopen.bmj.com/site/about/guidelines.xhtml

Sickle Eye Project: A cross-sectional, non-interventional study of the prevalence of visual impairment due to sickle cell retinopathy and maculopathy in the UK

Authors: Christiana Dinah[1,2]§, Konstantinos Balaskas[3], Brigit Greystoke[4], Rossby Awadzi[1], Peter Beke[5], Roger A'hern[6], James Talks[4]

§ **Corresponding author address:**

Christiana Dinah
Ophthalmology Department
London Northwest University Healthcare NHS Trust
Central Middlesex Hospital
London, United Kingdom
Email: christiana.dinah@nhs.net

Author affiliations:

[1] London North West University Healthcare NHS Trust
[2]Brain Sciences Department, Imperial College London
[3] NIHR Moorfields Biomedical Research Centre, Moorfields Eye Hospital NHS Foundation Trust, London, UK
[4] Royal Victoria Infirmary, Newcastle
[5]Lived experience expert
[6] Independent Statistician

Keywords: medical retina, anaemia, health services

Word count: 4899

For peer review only - http://bmjopen.bmj.com/site/about/guidelines.xhtml

ABSTRACT

Introduction

Sickle-cell disease (SCD) is one of the most common genetic disorders in the UK, with over 15000 people affected. Proliferative sickle cell retinopathy (SCR) is a well-described complication of SCD and can result in significant sight loss, although the prevalence in the UK is not currently known. There are currently no national screening guidelines for sickle cell retinopathy, with wide variations in the management of the condition across the UK.

Methods and analysis

The Sickle Eye Project is an epidemiological, cross-sectional, non-interventional study to determine the prevalence of visual impairment due to SCR and/or maculopathy in the UK. Haematologists in at least 16 geographically dispersed hospitals in the UK linked to participating eye clinics will offer study participation to consecutive patients meeting the inclusion criteria attending the sickle cell clinic. The following study procedures will be performed: a) best corrected visual acuity with habitual correction and pinhole b) dilated slit lamp biomicroscopy and funduscopy c) optical coherence tomography (OCT) d) OCT-angiography where available e) ultrawide fundus photography f) NEI-VFQ 25 g) acceptability of retinal screening questionnaire. The primary outcome is the proportion of people with sickle cell disease with visual impairment defined as LogMAR ≥0.3 in at least one eye. Secondary outcomes include the prevalence of each stage of sickle cell retinopathy and presence of maculopathy by age and genotype; correlation of stage of sickle cell retinopathy and maculopathy to severity of SCD, the impact of sickle cell retinopathy and presence of maculopathy on vision-related quality of life and the acceptability to patients of routine retinal imaging for sickle cell retinopathy and maculopathy.

Ethics and dissemination

Ethical approval was obtained from the **South Central – Oxford A Research Ethics Committee (REC** 23/SC/0363). Findings will be reported through academic journals in ophthalmology and haematology.

For peer review only - http://bmjopen.bmj.com/site/about/guidelines.xhtml
For peer review only

ARTICLE SUMMARY

Strengths and limitations of this study

- The Sickle Eye Project will involve a large, geographically diverse random sample of people with SCD attending routine haematology clinics, which is likely to be a representative sample of the SCD population in the UK.
- The study will assess the correlation between surrogates of severity of sickle cell disease and retinal imaging biomarkers
- The study will utilise modern retinal imaging and clinical examination, in addition to incorporating double-grading by expert graders and retinal specialists at Moorfields Reading Centre
- One limitation is that the prevalence data and the imaging findings pertain to people with sickle cell disease in the United Kingdom and may not be directly generalizable toother health systems, especially Africa and Asia, which are the continents with the highest prevalence of sickle cell disease.

For peer review only

BACKGROUND

Proliferative sickle cell retinopathy

Sickle-cell disease (SCD) is one of the most common genetic disorders in the UK, with approximately 15000 people affected (1). It is a debilitating, multisystem disease, associated with episodes of acute illness, progressive organ damage and reduced life expectancy. There are 13 genetic variants of SCD, with HbSS genotype being most common (>70%) and most severe, followed by HbSC genotype. SCD affects predominantly people of African and Afro-Caribbean descent and evidence demonstrates inequalities in healthcare outcomes and access for people with SCD (2). In the UK, an all-party parliamentary group inquiry reported low awareness of SCD and its complications among healthcare professionals and underinvestment in sickle cell research (3).

Proliferative sickle cell retinopathy (SCR) is the most common ophthalmic manifestation of SCD and can result in significant sight loss, although the prevalence in the UK is not currently known. Decades old retrospective and cohort studies from other countries have estimated prevalence of SCR in SCD patients at up to 48% and

For peer review only - http://bmjopen.bmj.com/site/about/guidelines.xhtml

associated with significant sight loss in up to 12% (4,5). Importantly, life expectancy for SCD has increased from 14 years in 1973 to >60 years in high-income countries with improvement in management and healthcare delivery (6). Evidence from cohort studies and large retrospective studies demonstrate increasing incidence of SCR with age (7,8). As such, we could expect the prevalence of SCR and potentially associated visual impairment, to be currently higher in the UK than previously reported.

Five stages of SCR were described by Goldberg in 1971 (9). These are progressive with stages 1-3 being asymptomatic, stage 3 reflecting the presence of retinal neovascularization in the far periphery known as seafan neovascularization, vitreous haemorrhage (bleeding inside the eye) develops in stage 4 and rhegmatogenous and/or tractional retinal detachment in stage 5.

Sickle cell maculopathy

In addition to the peripheral retina, SCD can also affect the macula (central retina) potentially causing visual loss, known as sickle cell maculopathy. Sickle cell maculopathy (SCM) is defined as patchy areas of severe retinal thinning in the temporal macula. The exact aetiology of macular thinning remains unclear, but it may be related to the temporal macula ending along the horizontal raphe, making it a watershed zone between the vascular arcades of the retinal circulation and thus more susceptible to vascular occlusions (10). Whilst high-contrast visual acuity was preserved in most patients demonstrated to have SCM in a single center study (11), irreversible scotoma, decreased retinal sensitivity and visual loss has been described in association with SCM (12,13,14). Maculopathy was not included in the Goldberg classification as its presence only became more apparent with modern imaging techniques. There is currently no treatment for vision loss due to maculopathy.

Current interventions for SCR and maculopathy

Laser photocoagulation is currently the only intervention with high-level evidence of safety and efficacy in the management of stage 3 SCR (15,16). Laser photocoagulation has an indirect effect as it destroys the ischaemic retina responsible for production of vascular endothelial growth factor (VEGF) that triggers the proliferation of new blood vessels. This technique is commonly used in the treatment of diabetic retinopathy and widely available in the UK However, it is not clear that the pathophysiology of sickle cell retinopathy is the same as diabetic retinopathy.

For SCR, there has been a paucity of randomized controlled trials (RCT) of intervention, with only 2 RCTs evaluating laser treatment for stage 3, and both conducted over 30 years ago. In the first randomized controlled trial of laser, Jampol et al (15) compared laser treatment to observation in a randomized controlled trial of 167 eyes and reported 3.4% developed vitreous haemorrhage (stage 4 SCR) in the laser-treated arm versus 27.5%

For peer review only - http://bmjopen.bmj.com/site/about/guidelines.xhtml

For peer review only

in the observation arm whilst 1% developed visual loss in the laser-treated arm versus 5% in the observation arm. Faber et al subsequently reported similarly positive results (16) with a Cochrane review of the safety and efficacy data of laser treatment for stage 3 SCR concluding that laser treatment reduces the risk of visual loss at mean follow-up of 1 year and is protective against the development of vitreous haemorrhage (17). However, the RCTs and other case series also reported spontaneous regression of stage 3 in up to 30% of eyes (7, 17). In addition, these RCTs evaluating laser treatment are more than 30 years old and laser technology as well as the systemic management of SCD has improved significantly in that time.

Alternatively, stage 3 and 4 SCR can be managed with intravitreal anti-vascular endothelial growth factor (anti-VEGF) therapy. Over the last 16 years, intravitreal anti-VEGF have become commonplace in ophthalmic practice with many licensed indications including age-related macular degeneration and diabetic retinopathy. As VEGF is implicated in SCR (18), the use of intravitreal anti-VEGF therapy is scientifically rational, and evidence of regression and resolution has been demonstrated in case series of eyes with stage 3 and 4 SCR (19). Additionally, angiopoietin-1 and angiopoietin-2 have also been implicated in the pathophysiology of SCR (20). In June 2022, NICE approved Faricimab, an intravitreal bi-specific monoclonal antibody that inhibits VEGF and angiopoietin-2 for the treatment of wet-age related macular degeneration and diabetic macular oedema (21,22). No RCTs have been conducted comparing intravitreal anti-VEGF therapy or anti-angiopoietin-2 to laser therapy or observation.

The main therapeutic intervention for stage 4 and 5 SCR is vitrectomy. Vitrectomy is a surgical intervention that involves removal of the vitreous (including blood if stage 4 SCR) and repositioning of the retina to its proper position against the back of the eye (when there is retinal detachment as in stage 5). Despite significant advances in modern surgical techniques, visual outcomes for stage 4 or 5 SCR remain poor, with final visual acuity post-surgery often significantly below driving vision (23), so early detection of SCR remains an opportunity to prevent sight loss.

There is also the potential for systemic treatment of SCD to influence the progression of SCR and/or maculopathy. Hydroxycarbamide is the gold standard treatment for prevention of painful crises in SCD and has been proven to reduce hospital admissions in patients with SCD (24). Hydroxycarbamide induces fetal haemoglobin production (HbF) thereby decreasing sickle haemoglobin erythrocyte polymers, haemolysis, and painful vaso-occlusive crisis. Rigorous investigation over the past 30 years has demonstrated the efficacy of hydroxycarbamide in reducing disease complications, health care utilization, and costs for patients with SCD. However, compliance rates are well established to be low and linked to a variety of reasons including fear of side effects, stigma and low health literacy (25). Although there are no prospective studies that evaluate the efficacy of hydroxycarbamide on reducing the incidence of SCR, large retrospective studies have highlighted

For peer review only - http://bmjopen.bmj.com/site/about/guidelines.xhtml

the protective effect of hydroxycarbamide on SCR and maculopathy (26,27). These studies suggest hydroxycarbamide, by elevating levels of HbF, can significantly reduce the odds of developing SCR and reduce the progression of sickle maculopathy. There is also emerging evidence that the severity of SCR and maculopathy correlates with severity of systemic anaemia associated with SCD (28,29). As such, retinal screening may improve compliance with hydroxycarbamide if evidence confirming protective retinal effects are corroborated. Retinal screening may also highlight the need for escalations of management to the haematologist.

Retinal imaging for SCR and maculopathy

Retinal imaging has undergone revolutionary advancement in the past 20 years. Ultrawide field fundus photography detects SCR more reliably than traditional eye examination or imaging (30). The widespread availability and use of optical coherence tomography (OCT) (and OCT-angiography in some centers), which are rapid, non-invasive imaging tools that produce detailed images of the macular and associated vascular plexuses means we can now detect damage to the macular otherwise undetected using traditional methods. These tools are now used routinely and extensively in the management of various high-volume eye diseases across the country including age-related macular degeneration, diabetic eye disease and patients with visual symptoms from SCR or maculopathy and are likely to be acceptable to all patients with SCR. The combination of ultra-widefield colour, OCT and OCTA will provide the best method for detecting vision-threatening retinal changes in sickle cell disease and detecting change over time.

Clinical study rationale

There is no reliable estimate of the burden of sight loss due to SCR and maculopathy in the UK or the number of people with different stages of SCR and maculopathy. This lack of data makes planning configuration and delivery of appropriate services for eye health in SCD difficult and could lead to unwarranted variation

Our study will determine the proportion of vision loss due to SCR and maculopathy in the UK. We will also determine the distribution of the stages of SCR and maculopathy and correlation with severity of SCD. SCR and maculopathy screening are easily incorporated into existing diagnostic clinic pathways for retinal diseases. However, there is a need to establish any benefit of screening and the acceptability to patients with SCD.

For peer review only - http://bmjopen.bmj.com/site/about/guidelines.xhtml

Study Objectives

The Sickle Eye Project includes an internal pilot phase, which is the first 4 months of the recruitment period, to assess recruitment and trial processes.

The objectives of the internal pilot are:

1. To open 6 sites by the end of the pilot phase
2. 95% of the expected monthly recruitment is achieved by the end of the pilot phase
3. Adherence to imaging protocol in ≥90% of cases
4. 95% of recruited participants with completed NEI VFQ 25 questionnaire

Main study primary objective: To determine the prevalence of visual impairment (best corrected visual acuity ≥LogMAR 0.3 in at least one eye) due to SCR and/or maculopathy in persons with SCD

Main study secondary objectives:

1. To determine the prevalence of each stage of SCR and maculopathy and correlation with:

i) number of hospital admissions with acute sickle cell episode in the past 12 months, ii) serum HbF level iii) serum Hb level

2. To determine the impact of SCR and maculopathy on vision-related quality of life.

3. To determine the acceptability to patients of retinal imaging and routine screening for SCR and maculopathy.

Outcome measures

Internal pilot outcomes

The following progression criteria using a red/amber/green traffic light system will be used to inform whether we progress to the main trial:

For peer review only

Site opening progress

Green: 6 sites are open to recruitment

Amber: 3 -5 sites are open to recruitment

Red: 2 or fewer sites or more are open to recruitment

Recruitment progress

Green: Recruitment of >120 at end of pilot phase

Amber: Recruitment of ≤ 120 but ≥ 79 patients at end of pilot phase

Red: Recruitment of <79 patients at end of pilot phase.

Fidelity check demonstrating adherence to image acquisition protocol

Green: Adherence to image acquisition protocol in ≥90% of cases.

Amber: Adherence to image acquisition protocol in ≥80%

Red: Adherence to image acquisition protocol in <80% of cases

Completion of the NEI VFQ-25

100% of recruited participants completing the NEI VFQ-25

Green: ≥95% of recruited participants with completed NEI VFQ-25

Amber: ≥80% of recruited participants with completed NEI VFQ-25

Red: <80% of recruited participants with completed NEI VFQ-25

If at the end of the pilot phase the thresholds are at amber or red for any of the progression decision points, the Co-Investigator Group, which includes a lived experience expert as co-applicant will review and provide remedial action that would allow progression to the main study to the Funder. If there are no obvious remedial actions that can be taken, the Co-Investigator group will discuss stopping the study with the Funder.

For peer review only - http://bmjopen.bmj.com/site/about/guidelines.xhtml

Main study primary outcome

1. The prevalence of visual impairment due to SCR or maculopathy (defined as best corrected visual acuity LogMAR ≥ 0.3 in at least one eye) in a representative sample of the UK population with SCD

Secondary outcomes

1. The prevalence of each stage of SCR and presence of maculopathy by age and genotype

2. The correlation of each stage of SCR and maculopathy to severity of SCD as determined by number of hospital admissions in the preceding 12 months/serum HbF/ serum Hb.

3. The impact of SCR and presence of maculopathy on vision-related quality of life.

4. The acceptability to patients of routine retinal imaging for SCR and maculopathy

5. The development of updated consensus definitions and a modernized classification system for SCR and maculopathy, leveraging the increased insight into retinal tissue anatomy and vascular blood flow afforded by contemporary imaging technologies

METHODS

This is a cross-sectional, non-interventional study, with planned recruitment over 15 months. Potential participants will be introduced to the study by their haematologist, who will also provide them with the participant information sheet. Identification of potential participants in the haematology clinic is important to prevent ascertainment bias and obtain a representative sample of people with SCD. All patients with SCD are offered regular specialist follow-up by a haematologist in the UK. The national haemoglobinopathy registry record for 2021 reported 12904 people with SCD confirming that an overwhelming proportion of SCD patients are under specialist care in the UK. Informed consent will be obtained from willing participants by the ophthalmology team either in the eye department or over the telephone. Informed consent will be obtained prior to the collection of any data. Once consented, participants will have best corrected visual acuity (BCVA) with habitual correction and pinhole measured, a slit lamp examination of the anterior segment and fundus, spectral-domain optical coherence tomography (SD-OCT), optical coherence tomography-angiography (OCT-A) and ultra-widefield fundus photography performed in the eye department. Subsequently, the participant will have the NEI VFQ-25 questionnaire and the acceptability of retinal screening questionnaire administered.

For peer review only - http://bmjopen.bmj.com/site/about/guidelines.xhtml

The NEI VFQ-25 questionnaire can be administered over the telephone either at the time of consent or after the visit to the eye department. The retinal screening questionnaire is to be administered to the participant once their retinal imaging studies have been performed.

If the NEI VFQ-25 is completed over the telephone, this must be done ±28 days of the eye clinic visit for examination and retinal images. The participant is subsequently asked if there have been any acute changes to their visual function.

The retinal images acquired from study participants will be uploaded to the Moorfields Reading Centre where they will be double graded with adjudication by a senior retinal specialist.

The research team will also complete an electronic case report form recording basic demographic history such as: Year of birth, Sex, Sickle genotype, Number of hospital admissions with acute sickle crisis, medical history, current medications and interventions for SCD, previous history of stage 4 or 5 sickle cell retinopathy, ocular comorbidities, previous ocular interventions and most recent serum haemoglobin (Hb) and serum haemoglobin F (HbF) within 3 months of study entry.

The planned study start date for participant recruitment is 14th of November 2023 and planned recruitment end date is 28th of February 2025

Patient and Public Involvement statement

Our patient and public involvement and engagement (PPIE) advisors, consisting of 16 people with lived experience of SCD have been involved in defining the research question and methodology for this study. The study management group also includes a co-applicant with lived experience of sickle cell disease who has been involved in the strategic management of the study including design of public engagement meetings, social media engagement with the public and during the recruitment phase will be involved in strategic decision-making for the effective delivery of the study. Our PPIE advisors informed us of the general lack of awareness of the potential effects of SCD on vision. They described the difficulties associated with vision loss including the increased vulnerability associated with loss of a driving license in the context of living with SCD, days off work associated with vitreous haemorrhage impacting their financial well-being and livelihoods and difficulty engaging in vision-related tasks at work and university. One of the members of our PPIE group told us they would have been compliant with their systemic medication if they knew it could help preserve their sight and the others agreed this could be a motivating factor for many people with SCD. This group will be involved in any changes to recruitment strategy and drafting the dissemination plan.

For peer review only - http://bmjopen.bmj.com/site/about/guidelines.xhtml

Participants and recruitment

Haematology teams in participating sites will inform consecutive patients with SCD ≥ 16 years who meet the study criteria about the study, providing them with the participant information sheet. They will refer interested participants to the ophthalmology unit for study participation. Participating Trusts have been selected as they already offer or are willing to offer routine eye screening for SCD patients as part of standard of care and confirmed to have ultra-widefield fundus imaging and spectral domain OCT. We have used the 2021 national haemoglobinopathy registry (NHR) report to identify centers treating patients with SCD in England. Eligible centers had to meet the following criteria:

1. At least 60 patients with SCD on the registry
2. Ultra-wide field fundus photography
3. Spectral domain OCT imaging
4. A routine screening programme for SCR in place or willing to institute one
5. Capacity and willingness to participate in the study

600 individuals who have been diagnosed at any point with SCD will be identified from up to 16 geographically dispersed participating NHS Trusts across the UK.

An invitation letter informing potential participants due to attend a sickle cell clinic can be sent out with appointment letters so that they are aware of the study before attending the clinic.

Interested participants will attend the eye clinic where they will be required to provide written informed consented and research activities will occur. For those who do not speak English or would benefit from translation services, this will be funded by the study. Informed consent can also be obtained over the telephone. Potentially eligible participants who have previously given consent to contact for any research can be approached by the research team by telephone and if willing to consider participating, the patient information sheet (PIS) can be posted to them. There will be an opportunity to discuss the study before consent is obtained in the eye clinic or by telephone, and it will be made clear that the individual is free to withdraw from the study at any point.

Eligibility criteria:

Inclusion criteria

- Willingness to participate

For peer review only - http://bmjopen.bmj.com/site/about/guidelines.xhtml

- Ability to provide informed consent
- Age 16 years or older
- Diagnosis of SCD of any genotype

Exclusion criteria

- Inability to consent
- Poor image quality
- Age <16 years
- Sickle cell trait only

Study activities

A summary of the schedule of activities is listed in Table 1

Theoretical framework of acceptability (TFA)-based acceptability questionnaire

The theoretical framework of acceptability (TFA) was developed in response to recommendations that acceptability should be assessed in the design, evaluation and implementation phases of healthcare interventions (31). The TFA consists of seven component constructs (affective attitude, burden, ethicality, intervention coherence, opportunity costs, perceived effectiveness, and self-efficacy) that can help to identify characteristics of interventions that may be improved. Using this robust framework, a validated generic TFA-based questionnaire (32) was developed as an adaptable tool to measure acceptability across various healthcare interventions.

Pre-validating the questionnaire for acceptability of routine retinal screening in persons with SCD

We have adapted the generic TFA questionnaire with insights from our PPI group pilot, expert clinical opinion and literature review. The first draft of the adapted TFA questionnaire has been discussed with 10 members of our PPIE group. This feedback was incorporated and shared with a further group of 10 patients attending retinal screening clinics for SCD at 2 eye units in the UK to check comprehensibility and comprehensiveness. The final version of the questionnaire incorporates this input from patients and medical experts managing patients with SCD.

For peer review only - http://bmjopen.bmj.com/site/about/guidelines.xhtml

Other study activities

Best corrected visual acuity (BCVA) with habitual correction and pinhole

This is a measure of the primary outcome. BCVA will be assessed using the Early Treatment of Diabetic Retinopathy Study vision chart in a 4-meter lane under routine clinic conditions. Participants will be invited to bring their most up to date correction to the study visit. BCVA will be recorded with their habitual correction and pinhole in LogMAR. **Refraction by an optometrist is not required**

Slit lamp biomicroscopy and dilated funduscopy

Slit lamp biomicroscopy allows the ophthalmologist to microscopically examine the eye for any abnormalities or problems. Dilated funduscopy allows better evaluation of the lens and vitreous. This will be performed to document other ocular causes of visual impairment such as corneal scars, cataracts and vitreous haemorrhage.

Spectral domain OCT

Spectral domain OCT (SD-OCT) is the current reference standard for diagnosing and monitoring progression of various macular diseases. In sickle cell maculopathy, there is evidence of retinal thinning, with the temporal macular being most commonly affected.

OCT angiography

OCT-Angiography (OCT-A) provides depth-resolved visualization of the superficial and deep capillary plexuses. By allowing depth-resolved visualization of the macular vascular network with high resolution, optical coherence tomography angiography (OCTA) recently detected much more macular vascular alterations than previously detected using just OCT and provided additional features, including enlargement and irregularities of the foveal avascular zone (FAZ), hairpin venular loops, and areas of flow loss responsible for irreversible macular thinning (33).

Ultra-widefield fundus photography

Ultra-widefield fundus photography captures an image covering more than 80% of the retina. It allows the observer to assess the retina, associated blood vessels and optic nerve. This is particularly of benefit in sickle

For peer review only - http://bmjopen.bmj.com/site/about/guidelines.xhtml

cell retinopathy, where the initial stages of proliferative retinopathy occur in the far periphery and can be missed if the periphery is not carefully assessed. All units included in this study will be using either the Optos or the Clarus. Whilst there is evidence that un-dilated ultrawide field fundus photography can be of utility in screening for sickle cell retinopathy with a specificity of 100% compared to dilated funduscopy examination in one study, the sensitivity was 69.2% (34). Therefore, for the purpose of this study, ultra-widefield fundus photography will be performed with a dilated pupil.

NEI-VFQ 25

The NEI-VFQ 25 is a validated 25 item questionnaire comprising of 11 subscales on different aspects of vision-related functioning (VRQoL), quality of life and 1 item on general health.

Due to the proliferation of diagnostic hubs in the NHS, the NEI-VFQ-25, which can take up to 15 minutes to complete may limit recruitment at some sites. As such, we will monitor the impact of completing this questionnaire on recruitment through our monthly investigators meeting for the first 3 months of recruitment. Feedback from Principal Investigators on the impact of the NEI-VFQ 25 on recruitment will determine whether to switch to the EQ-5D with vision bolt-on.

EQ-5D with vision bolt-on

The 5 level EQ-5D is a widely used preference-based health related quality of life instrument with one question for each of 5 core dimensions of health: mobility, self-care, usual activities, pain/discomfort and anxiety/depression. To capture the effect of conditions that affect sensory functions or cognition that may not be adequately captured by the instrument, additional dimensions, known as ‘bolt-ons’ were developed. The vision bolt-on has demonstrated enhanced sensitivity, construct validity and responsiveness to change in populations with vision problems (35,36). Importantly, EQ-5D with vision bolt-on comprises of 6 questions which can be rapidly completed by participants.

For peer review only - http://bmjopen.bmj.com/site/about/guidelines.xhtml

Study activity	Personnel	Baseline	±28 days
**Eligibility criteria check**	Haematology team	x	
**Patient information sheet**	Haematology team	x	
**Informed consent**	Ophthalmology team	x (Can be completed by telephone)	
**BCVA with habitual correction and pinhole**	Ophthalmology team	x	
**Slit lamp biomicroscopy and dilated funduscopy**	Ophthalmology team	x	
**OCT**	Ophthalmology team	x	
**OCT-A**	Ophthalmology team	x	
**Ultrawide field fundus photography**	Ophthalmology team	x	
***NEI-VFQ 25**	Ophthalmology team	x (Can be completed by telephone)	x

For peer review only - http://bmjopen.bmj.com/site/about/guidelines.xhtml

***Acceptability Questionnaire**	Ophthalmology team	x (Can be completed by telephone after retinal imaging and within 14 days)	x
**Clinical assessment of images**	Ophthalmology	x	
**Completion of case report form**	Ophthalmology/ haematology team	x	x
**Transfer of images**	Ophthalmology team	x	x
**Grading of images**	Moorfields Reading Centre		Performed in batches throughout the study period

Table 1: Schedule of activities, study personnel responsible and study timeline

Image acquisition protocol and transfer of data

Imaging acquisition will be performed by trained staff at each recruitment site. As this is a pragmatic study, and the imaging to be acquired are standard of care in NHS eye departments, standards for training and experience of staff performing the imaging acquisition function will be the ones applicable at each recruitment site as per local Standard Operating Procedures.

The Moorfields Reading Centre will provide supporting material of educational and advisory character for acquisition of high-quality imaging from the Sickle Eye Project participants. It will also provide detailed, yet simple and intuitive instructions for imaging file export and direct upload to the Moorfields Grading Portal Web-based Application.

For peer review only - http://bmjopen.bmj.com/site/about/guidelines.xhtml

For each patient, imaging from the three following modalities will be obtained, and transferred to the Moorfields Reading Centre. Imaging from both eyes will be obtained:

- Optical Coherence Tomography scan (TopCon 3D-2000, Heidelberg Spectralis or Zeiss Cirrus). Recommended parameters: Macula Centre 6x6 mm Raster Scans.

- Optical Coherence Tomography Angiography where available (TopCon Triton, Heidelberg Spectralis OCT-2, Zeiss Angioplex)

Recommended parameters: Macula Centre 6x6 OCT Angiography mode

- Wide Field Imaging (Optos California or Zeiss Clarus)
Recommended parameters for wide field imaging: Instructions will be provided to assist imaging acquisition staff with obtaining clear view of the Fundus, including the extreme periphery through imaging acquisition at the central and 4 peripheral positions of gaze (up, down, right, left) and the avoidance of 'eyelash' artifacts that interfere with the fundalundus view in Optos Ultra-Wide Field Imaging.

The images being transferred to Moorfields Reading Centre will be link-anonymized with no patient identifiers. The link key will remain with each recruiting site and will not be shared with the sponsor site.

Bi-directional communication between recruitment sites and the Moorfields Reading Centre will be possible through the interactive Moorfields Grading Portal. A granular Audit Log will record all study-related activity, including who, where and when each activity was performed.

For peer review only

STATISTICS AND DATA ANALYSIS

The primary analysis will be to calculate the prevalence of sickle cell retinopathy/maculopathy-related vision loss together with its 95% confidence interval (CI) to illustrate the uncertainty in the estimate. It is anticipated the prevalence will be approximately 10% based on data from Saidkasimova et al (37). With 554 subjects the prevalence can be estimated with a 95% confidence interval with a 5% width e.g. expected to be from 7.5% to 12.5%. The sample size (N) was chosen to satisfy this 5% width, given the confidence interval is expected to be p +/- 1.96*sqrt(p*(1-p)/n), where p=0.1. To obtain this number of evaluable subjects we estimate approximately 600 subjects will have to be enrolled, this allows for a drop-out rate of just under 10%.

For peer review only - http://bmjopen.bmj.com/site/about/guidelines.xhtml

A sample size of 600 will also allow accurate estimates of the stages of SCR and maculopathy occurrence to be obtained. For example, the CIs in Table 2 are based on a French retrospective cohort of 942 patients (8) :

	%	N	95%CI	Width
**Stage 0**	30%	166	(23%-37%)	14%
**Stage 1-2**	40%	222	(34%-46%)	13%
**Stage 3**	20%	110	(13%-27%)	15%
**Stage 4**	5%	28	(8%-20%)	12%
**Stage 5**	5%	28	(8%-20%)	12%
	100%	554		

Table 2: Confidence intervals from large retrospective study of sickle cell retinopathy in France

Other endpoints also include the calculation of the prevalence of each stage of SCR by age bracket and genotype. Sample size relevance in these circumstances can be illustrated by considering a hypothetical outcome. For example, under the null hypothesis there would be no association between prevalence and age, and given the hypothetical distributions of stage and age below (Table 3), it can be shown that the estimated correlation (Spearman Rank) has a greater than 3 SE difference (from zero) with 554 cases. This suggests powering is adequate for associations of this size, by the 'rule of three', for 85% power (Z beta=1) and 5% sig level (Zalpha=1.96).

Age Group	***rate overa ll***

For peer review only - http://bmjopen.bmj.com/site/about/guidelines.xhtml

**N**		*3.1%*	*7.4%*	*8.2%*	*14.0%*	*16.9%*	*21.7%*	***9.7%***
	**Stage/Age**	**16-25**	**26-35**	**36-45**	**46-55**	**56-65**	**66+**	
	5	1	5	6	6	5	2	
**SCR**	4	2	5	5	8	6	3	
	3	23	28	28	20	12	4	
	1-2	46	56	55	39	23	8	
	0	24	42	40	27	19	6	
		96	*136*	*134*	*100*	*65*	*23*	554

Spearman Rank -0.14 (95%CI:-0.23 to -0.06), p=0.001

Table 3: Hypothetical distribution of stage of sickle cell retinopathy and age

Data Analysis plan

The primary analysis is to estimate the prevalence of SCD-related vision loss. In addition to the calculated value, a corresponding 95% confidence interval will be obtained to illustrate its associated uncertainty. We will also calculate the prevalence of each stage of SCR and maculopathy by age bracket and genotype. The unit of analysis will be the eye with the worse SCR stage, or randomly chosen eye if both are of the same stage. The chi-square test for trend will be used if groups are ordinal. The Kruskal-Wallis test (categorical factors) or Spearman rank (ordinal factors) will be used when comparing SCR stages between groups, as above.

The association between SCD severity (as assessed by number of hospital admissions in past year and haemoglobin and haemoglobin F level within 3 months) with SCR stage and presence of sickle maculopathy will be examined using Spearman Rank or Pearson product correlation. Multivariable analysis, such as logistic regression, will be undertaken to assess the simultaneous effects of multiple factors on stage of SCR and presence of maculopathy. The correlation between NEI VFQ-25 global score and acceptability with best corrected visual acuity, SCR stage and presence of maculopathy will also be assessed using Spearman rank correlation.

For peer review only - http://bmjopen.bmj.com/site/about/guidelines.xhtml

ETHICS AND DISSEMINATION

Ethics approval

The project will meet the requirements and principles set by General Data Protection Regulations 2018 and the European Medicines Agency (GCP Guidelines).

No study activity will take place prior to all the approvals being in place from the relevant agencies, and permission to carry out the research has been received from the local R&I office.

The study received Health Research Authority and Health and Care Research Wales Approval, REC reference: 23/SC/0363 on the 6th of October 2023 and will receive local research and development (R&D) capacity and capability at participating sites. The study will be conducted in accordance with the recommendations for physicians involved in research on human subjects adopted by the 18th World Medical Assembly, Helsinki 1964 (incl. later revisions) and any other relevant ethical guidance. Any subsequent changes to the study conduct, design or management will be notified to original approving R&D Department and any other relevant regulatory authority via a substantial amendment.

PUBLICATION AND DISSEMINATION POLICY

Results of this study will be submitted for publication in high-impact peer reviewed Ophthalmology and haematology journals. The manuscript will be prepared by the CI and authorship will be determined by the Sickle Eye Project publication policy. The results will also be presented at conferences such as the Royal College of Ophthalmologists Annual Congress, the British Society of Haematologists. The study question has the potential to influence the eyecare landscape for people with Sickle Cell Disease, promote closer working relationships and collaboration between haematologists and ophthalmologists and support further research into the utilization of retinal images in the management and assessment of treatment response in Sickle Cell Disease. This view has been validated by the health professionals and extensive patient engagement work across the country. Methods to ensure rapid clinical impact once the results are available include: submitting the study findings to NHS England and the Scientific committee in charge of generating guidelines by the Royal College of Ophthalmologists, production of lay information for dissemination via patient charities and organizations.

ACKNOWLEDGMENTS

We would like to thank the sixteen members of our Sickle Cell Peer Advisory group, for their time and invaluable input into the study so far.

For peer review only - http://bmjopen.bmj.com/site/about/guidelines.xhtml

Author Contributions

CD, JT and KB conceptualized the project; CD,JT,KB,RA,BG,PB and RA'H contributed to the study design, methodology, recruitment plan. CD prepared the initial draft of the manuscript; CD,JT,KB,RA,BG,PB and RA'H critically reviewed, edited and finalized the manuscript. CD, JT,KB,RA,BG,PB and RA'H have read and approved the final manuscript

Funding statement

This work has been supported by NIHR RFPB Grant (Ref: NIHR204961) and an unrestricted grant from Roche UK (Ref:M-GB-00010028) . The views expressed are those of the authors and not necessarily those of the NIHR, the department of health and social care or Roche UK.

Competing interest statement

CD reports receiving institutional research grant, consulting fees and speaker fees from Roche, KB has received consulting and speaker fees from Roche and JT has received consulting and speaker fees from Roche. The other authors have no competing interests to declare

REFERENCES

1. Dormandy E, James J, Inusa B, et al. How many people have sickle cell disease in the UK? J Public Health (Oxf) 2018;40(3):e291-e295.
2. Aljuburi G, Laverty AA, Green SA, et al. Socio-economic deprivation and risk of emergency readmission and inpatient mortality in people with sickle cell disease in England: observational study. J Public Health (Oxf) 2013;35(4):510-7.
3. Sickle Cell Society. Sickle cell disease: no one is listening. 2021. [Online]. Available at: https://www.sicklecellsociety.org./wpcontent/uploads/2021/11/No-ones-listening-final.pdf. [Accessed 11 November 2021].
4. Moriarty BJ, Acheson RW, Condon PI, et al. Patterns of visual loss in untreated sickle cell retinopathy. Eye (Lond) 1988;2(Pt 3):330-5.
5. Condon PI, Serjeant GR. Behaviour of untreated proliferative sickle retinopathy. Br J Ophthalmol 1980;64(6):404-11.
6. Gardner K, Douiri A, Drasar E, et al. Survival in adults with sickle cell disease in a high-income setting. Blood 2016;128(10):1436-8. doi: 10.1182/blood-2016-05-716910.
7. Downes SM, Hambleton IR, Chuang EL, et al. Incidence and natural history of proliferative sickle cell retinopathy: observations from a cohort study. Ophthalmology 2005;112(11):1869-75.

For peer review only - http://bmjopen.bmj.com/site/about/guidelines.xhtml

8. Leveziel N, Bastuji-Garin S, Lalloum F, et al. Clinical and laboratory factors associated with the severity of proliferative sickle cell retinopathy in patients with sickle cell hemoglobin C (SC) and homozygous sickle cell (SS) disease. Medicine (Baltimore) 2011;90(6):372-378.
9. Goldberg MF. Classification and pathogenesis of proliferative sickle cell retinopathy. Am J Ophthalmol 1971;71(3):649-65.
10. Stevens TS, Busse B, Lee CB, et al. Sickling hemoglobinopathies; macular and perimacular vascular abnormalities. Arch Ophthalmol 1974;92(6):455-63.
11. Sahak H, Saqalain, Lott P, et al. Sickle cell maculopathy: prevalence, associations and impact on visual acuity. Ophthalmologica 2021;244(2):159-164.
12. Lee CM, Charles HC, Smith RT, et al. Quantification of macular ischaemia in sickle cell retinopathy. Br J Ophthalmol 1987;71(7):540-545.
13. Kwok JM, Jeeva-Patel T, Margolin EA. Binasal visual field defects due to sickle cell maculopathy. J Neuroophthalmol 2022;42(1):e434-e436.
14. Chow CC, Genead MA, Anastasakis A, et al. Structural and functional correlation in sickle cell retinopathy using spectral domain optical coherence tomography and scanning laser ophthalmoscope microperimetry. Am J Ophthalmol 2011;152(4):704-711.e2.
15. Jampol LM, Condon P, Farber M, et al. A randomized clinical trial of feeder vessel photocoagulation of proliferative sickle cell retinopathy. I. Preliminary results. Ophthalmology 1983;90(5):540-5.
16. Farber MD, Jampol LW, Fox P, et al. A randomised clinical trial of scatter photocoagulation of proliferative sickle cell retinopathy. Arch Ophthalmol 1991;109(3):363-7.
17. Myint KT, Sahoo S, Thein AW, et al. Laser therapy for retinopathy in sickle cell disease. Cochrane Database Syst Rev 2015;(10):CD010790.
18. Cao J, Mathews MK, McLeod DS, et al. Angiogenic factors in human proliferative sickle cell retinopathy. Br J Ophthalmol 1999;83(7):838-46.
19. Cai CX, Linz MO, Scott AW. Intravitreal bevacizumab for proliferative sickle retinopathy: a case series. J Vitreoretin Dis 2018;2:32-38.
20. Mohan JS, Lip PL, Blann AD, et al. The angiopoietin/Tie-2 system in proliferative sickle cell retinopathy: relation to vascular endothelial growth factor, its soluble receptor Flt-1 and von Willebrand factor, and to the effects of laser treatment. Br J Ophthalmol 2005;89(7):815-9.
21. National Institute for Health and Care Excellence (NICE). Faricimab for treating wet age-related macular degeneration. [TA800]. 2023. Available at: https://www.nice.org.uk/guidance/ta800.
22. National Institute for Health and Care Excellence (NICE). Faricimab for treating diabetic macular oedema. [TA799]. 2023. Available at: https://www.nice.org.uk/guidance/ta799.

For peer review only - http://bmjopen.bmj.com/site/about/guidelines.xhtml

23. Ho J, Grabowska A, Ugarte M, et al. A comparison of 23 gauge and 20 gauge vitrectomy for proliferative sickle cell retinopathy – clinical outcomes and surgical management. Eye (Lond) 2018;32(9):1449-1454.
24. Nevitt SJ, Jones AP, Howard J. Hydroxyurea (hydroxycarbamide) for sickle cell disease. Cochrane Database Syst Rev 2017;(4):CD002202.
25. Adams-Graves P, Bronte-Jordan L. Recent treatment guidelines for managing adult patients with sickle cell disease: challenges in access to care, social issues, and adherence. Expert Rev Hematol 2016;9(6):541-52.
26. Mian UK, Tang J, Allende APM, et al. Elevated fetal haemoglobin levels are associated with decreased incidence of retinopathy in adults with sickle cell disease. Br J Haematol 2018;183(5):807-811.
27. Lim JI, Niec M, Sun J, et al. Longitudinal Assessment of Retinal Thinning in Adults With and Without Sickle Cell Retinopathy Using Spectral-Domain Optical Coherence Tomography. JAMA Ophthalmol 2021;139(3):330-337.
28. Duan XJ, Lanzkron S, Linz MO, et al. Clinical and ophthalmic factors associated with the severity of sickle cell retinopathy. Am J Ophthalmol 2019;197:105-113.
29. Fares S, Hajjar S, Romana M, et al. Sickle cell maculopathy: microstructural analysis using OCTA and identification of genetic, systemic and biological risk factors. Am J Ophthalmol 2021;224:7-17.
30. Alabduljalil T, Cheung S, Vandenhoven C, et al. Retinal ultra-wide field colour imaging versus dilated fundus examination to screen for sickle cell retinopathy. Br J Ophthalmol 2021;105:1121-6.
31. Sekhon M, Cartwright M, Francis J. Acceptability of healthcare interventions: an overview of reviews and development of a theoretical framework. BMC Health Serv Res 2017;17(1):88.
32. Sekhon M, Cartwright M, Francis JJ. Development of a theory-informed questionnaire to assess the acceptability of healthcare interventions. BMC Health Serv Res 2022;22(1):279.
33. Guerra R, Guerra C, Bastos M, et al. Sickle cell retinopathy: what we now understand using optical coherence tomography angiography. A systematic review. Blood Rev 2019;35:32-42.
34. Ahmed I, Pradeep T, Goldberg M, et al. Nonmydriatic ultra-widefield fundus photography in a hematology clinic shows utility for screening of sickle cell retinopathy. Am J Ophthalmol 2022;236:241-248.
35. Luo N, Wang X, Ang M, et al. A vision 'bolt-on' item could increase the discriminatory power of the EQ-5D index score. Value Health 2015;18(8):1037-1042.
36. Gandhi M, Ang M, Teo K, et al. A vision 'bolt-on' increases the responsiveness of EQ-5D: preliminary evidence from a study of cataract surgery. Eur J Health Econ 2020;21(4):501-511.
37. Saidkasimova S, Shalchi Z, Mahroo OA et al. Risk factors for visual impairment in patients with sickle cell disease in London. Eur J Ophthalmol. 2016;26(5):431-5.

For peer review only

For peer review only - http://bmjopen.bmj.com/site/about/guidelines.xhtml

For peer review only

For peer review only - http://bmjopen.bmj.com/site/about/guidelines.xhtml

STROBE Statement—Checklist of items that should be included in reports of ***cross-sectional studies***

	Item No	Recommendation	Page No
**Title and abstract**	1	(*a*) Indicate the study's design with a commonly used term in the title or the abstract	1-2
		(*b*) Provide in the abstract an informative and balanced summary of what was done and what was found	2
**Introduction**			
Background/rationale	2	Explain the scientific background and rationale for the investigation being reported	3-6
Objectives	3	State specific objectives, including any prespecified hypotheses	7
**Methods**			
Study design	4	Present key elements of study design early in the paper	9
6Setting	5	Describe the setting, locations, and relevant dates, including periods of recruitment, exposure, follow-up, and data collection	9
Participants	6	(*a*) Give the eligibility criteria, and the sources and methods of selection of participants	11
Variables	7	Clearly define all outcomes, exposures, predictors, potential confounders, and effect modifiers. Give diagnostic criteria, if applicable	13-14
Data sources/ measurement	8*	For each variable of interest, give sources of data and details of methods of assessment (measurement). Describe comparability of assessment methods if there is more than one group	*13-16*
Bias	9	Describe any efforts to address potential sources of bias	9
Study size	10	Explain how the study size was arrived at	17
Quantitative variables	11	Explain how quantitative variables were handled in the analyses. If applicable, describe which groupings were chosen and why	17-19
Statistical methods	12	(*a*) Describe all statistical methods, including those used to control for confounding	17-19
		(*b*) Describe any methods used to examine subgroups and interactions	17-19
		(*c*) Explain how missing data were addressed	
		(*d*) If applicable, describe analytical methods taking account of sampling strategy	17-19
		(*e*) Describe any sensitivity analyses	
**Results**			
Participants	13*	(a) Report numbers of individuals at each stage of study—eg numbers potentially eligible, examined for eligibility, confirmed eligible, included in the study, completing follow-up, and analysed	
		(b) Give reasons for non-participation at each stage	
		(c) Consider use of a flow diagram	
Descriptive data	14*	(a) Give characteristics of study participants (eg demographic, clinical, social) and information on exposures and potential confounders	
		(b) Indicate number of participants with missing data for each variable of interest	
Outcome data	15*	Report numbers of outcome events or summary measures	

For peer review only

Main results	16	(*a*) Give unadjusted estimates and, if applicable, confounder-adjusted estimates and their precision (eg, 95% confidence interval). Make clear which confounders were adjusted for and why they were included	
		(*b*) Report category boundaries when continuous variables were categorized	
		(*c*) If relevant, consider translating estimates of relative risk into absolute risk for a meaningful time period	
Other analyses	17	Report other analyses done—eg analyses of subgroups and interactions, and sensitivity analyses	
**Discussion**			
Key results	18	Summarise key results with reference to study objectives	
Limitations	19	Discuss limitations of the study, taking into account sources of potential bias or imprecision. Discuss both direction and magnitude of any potential bias	
Interpretation	20	Give a cautious overall interpretation of results considering objectives, limitations, multiplicity of analyses, results from similar studies, and other relevant evidence	
Generalisability	21	Discuss the generalisability (external validity) of the study results	
**Other information**			
Funding	22	Give the source of funding and the role of the funders for the present study and, if applicable, for the original study on which the present article is based	

*Give information separately for exposed and unexposed groups.

Note: An Explanation and Elaboration article discusses each checklist item and gives methodological background and published examples of transparent reporting. The STROBE checklist is best used in conjunction with this article (freely available on the Web sites of PLoS Medicine at http://www.plosmedicine.org/, Annals of Internal Medicine at http://www.annals.org/, and Epidemiology at http://www.epidem.com/). Information on the STROBE Initiative is available at www.strobe-statement.org.

For peer review only
