# Supplementary material for: Sickle Eye Project: a cross-sectional, non-interventional study of the prevalence of visual impairment due to sickle cell retinopathy and maculopathy in the UK
Source: BMJ Open. 2024 Feb 28;14(2):e082471. doi: 10.1136/bmjopen-2023-082471 (PMC10910489; doi:10.1136/bmjopen-2023-082471)
Supplement: Reviewer comments [file bmjopen-2023-082471.reviewer_comments.pdf]

PEER REVIEW HISTORY

BMJ Open publishes all reviews undertaken for accepted manuscripts. Reviewers are asked to complete a checklist review form (**http://bmjopen.bmj.com/site/about/resources/checklist.pdf**) and are provided with free text boxes to elaborate on their assessment. These free text comments are reproduced below.

ARTICLE DETAILS

**TITLE (PROVISIONAL)**	Sickle Eye Project: A cross-sectional, non-interventional study of the prevalence of visual impairment due to sickle cell retinopathy and maculopathy in the UK
**AUTHORS**	Dinah, Christiana; Balaskas, Konstantinos; Greystoke, Brigit; Awadzi, Rossby; Beke, Peter; Ahern, Roger; Talks, S.

This article was not externally reviewed at BMJ Open. Protocols that have been independently assessed prior to submission to BMJ Open are usually fast-tracked to publication on the grounds that further substantial changes will not be possible. This independent assessment will usually be external, independent review for both a substantial grant award from a non-commercial or government funder and ethics approval.
